# Neutral buffered electrolytes guarantee ideal band-edge pinning for semiconductor photoanodes†

**DOI:** 10.1039/d5sc01816a

**Published:** 2025-07-10

**Authors:** Yosuke Kageshima, Hiromu Kumagai, Katsuya Teshima, Kazunari Domen, Hiromasa Nishikiori

**Affiliations:** a Department of Materials Chemistry, Faculty of Engineering, Shinshu University 4-17-1 Wakasato Nagano 380-8553 Japan kage_ysk@shinshu-u.ac.jp nishiki@shinshu-u.ac.jp; b Research Initiative for Supra-Materials (RISM), Shinshu University 4-17-1 Wakasato Nagano 380-8553 Japan; c Research Center for Advanced Science and Technology, The University of Tokyo 4-6-1 Komaba Meguro-ku Tokyo 153-8904 Japan; d Office of University Professors, The University of Tokyo 7-3-1 Hongo Bunkyo-ku Tokyo 113-8656 Japan

## Abstract

The photoelectrochemical (PEC) processes occurring in semiconductor photoelectrodes have been regarded as similar to the physical processes occurring at the semiconductor/metal interfaces. In contrast, the physicochemical processes occurring in the electrolyte have been considered to be unaffected by the (photo)electrode materials that are employed. We found that these “ideal” situations are not always guaranteed during performing the actual PEC reaction. That is, the present study based on impedance and hydrodynamic voltammetry analyses proposes that the band diagram at the interface between a semiconductor photoanode and an electrolyte can be affected by transient physicochemical phenomena in the electrolyte during the PEC oxygen evolution reaction. Specifically, in the case that a neutral unbuffered electrolyte was employed, a local pH gradient was formed during the reaction and produced a positive shift in the flat-band potential. This means the breakdown of the ideal band bending at the Schottky-like junction. Meanwhile, a neutral buffered phosphate-based electrolyte suppressed the formation of this pH gradient and thus guaranteed ideal band-edge pinning at the photoanode/electrolyte interface. This study provides insights demonstrating that PEC water splitting occurring at the semiconductor/electrolyte interface are distinct from simple analogy to the conventional semiconductor physics and to the physicochemical processes in the electrolyte.

## Abstract 2

The band diagram at the photoanode/electrolyte interface can be affected by transient physicochemical phenomena in the electrolyte during O_2_ evolution. Buffered electrolytes suppress a pH gradient and thus guarantee an ideal Schottky-like junction.

## Graphical abstract

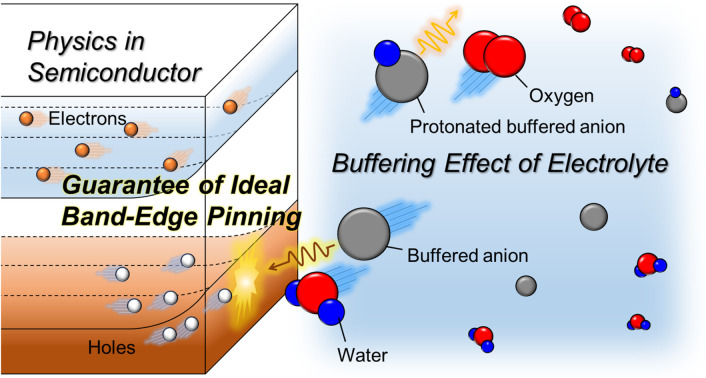

## Introduction

Ongoing interest in artificial photosynthesis has spurred research into photoelectrochemical (PEC) water splitting systems.^1^ The behaviour of the semiconductor electrodes that may be used during such processes in electrolytes has been explained by analogy to the conventional physics related to the characteristics of interfaces between semiconductors and metals or of photovoltaic systems.^2^ Specifically, in the case that an n-type semiconductor and an electrolyte are in contact and reach equilibrium under dark conditions, the valence and conduction bands of the semiconductor at the interface bend, with the band edges being pinned at specific potentials. An example of this scenario is the Schottky barrier junction formed at the interface between a semiconductor and a metal. Papers on pH dependence of photoelectrodes have established that the flat-band potential is determined by equilibrium of chemical species between the photoelectrode surface and the bulk electrolyte.^3–6^ That is, the surface of a metal oxide semiconductor is positively charged by the protonation at pH < the point of zero charge (PZC) or is negatively charged by the deprotonation at pH > PZC, resulting in the Nernst-like shift of the flat-band potential depending on the bulk electrolyte pH.^7^ It has been believed that, once the band-edge potentials are determined by the bulk electrolyte concentration, the band position should always be fixed even when the photoelectrode is performing PEC reaction under an applied potential and a light illumination. In this scenario, the performance of a photocatalyst or a photoelectrode material has typically been considered to be dependent mainly on the characteristics of the semiconductor, such as the band structure, the presence of electronic defect levels and the surface adsorption capability, irrespective of the electrolyte that is used.

The utilization of a buffered aqueous electrolyte can enable effective water electrolysis even at near-neutral pH values.^8–10^ This is possible because the buffering anions accelerate proton-based mass transfer in the electrolyte in the vicinity of the electrocatalyst during the hydrogen and oxygen evolution reactions. Another important contribution of the buffered electrolyte is the suppressed formation of a local pH gradient.^11–16^ Buffered electrolytes have sometimes also been employed to assess semiconductor photoelectrodes.^17–19^ The kinetics of physicochemical phenomena occurring in a liquid medium (*i.e.*, the diffusion of reactants and pH gradient) have been considered to be primarily determined by the electrolyte itself, irrespective of whether an electrocatalyst or semiconductor photoelectrode is employed. Hence, although the voltage losses inside the electrolyte have been well established in many experimental and theoretical studies,^8–16^ the interaction between the physical properties of the semiconductor and the physicochemical phenomena in the electrolyte was never considered.

In many cases, it has been assumed that the individual characteristics of the photoelectrode and the electrolyte cause separate phenomena to occur, but is this really the case? Since at the very least, electrolytes and metals, as well as photoelectrodes and electrocatalysts, are different, can the conventional physics of the semiconductor/metal interface or the physical chemistry in the electrolyte alone explain all PEC phenomena occurring at this interface? The present study elucidated the mechanism by which the buffering effect of the electrolyte affects the PEC oxygen evolution performance of a photoanode. The results showed that reduction of the local pH gradient close to the photoanode surface by the buffering effect of a neutral phosphate solution guaranteed ideal band-edge pinning, not limited to the well-known effect of the suppressed concentration overpotential inside the electrolyte. This is the first experimental demonstration that the electronic band structure at the solid/liquid interface can be affected by the transient physicochemical phenomena in the electrolyte.

## Results and discussion

### Unique PEC behaviour depending on electrolytes

**Fig. 1:**
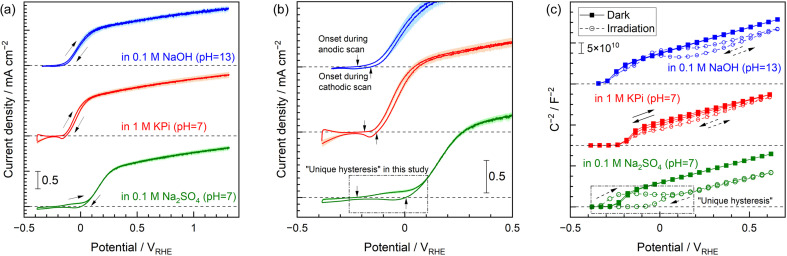
(a) CV data obtained using a Nb:STO single-crystal photoanode in various electrolytes upon exposure to a 365 nm LED and applying a scan rate of 10 mV s^−1^. Each electrolyte was purged with Ar and vigorously stirred. The photocurrent values reported here were average of three times of individual CV measurements, and the shaded areas around each plot indicate the standard error. (b) CV data in the vicinity of the onset potentials. (c) Mott–Schottky plots acquired in the absence of forced convection using a magnetic stirring bar with and without UV irradiation, applying a frequency of 5 kHz to the photoanode with a 10 mV amplitude. The arrows in (a and c) indicate the potential scan direction whereas those in (b) show the apparent onset potentials.

Cyclic voltammograms (CVs) obtained from a single-crystal Nb-doped SrTiO_3_ (Nb:STO) photoanode in various electrolytes with forced convection of vigorous stirring are presented in Fig. 1a. When a 0.1 M NaOH or 1 M potassium phosphate (KPi) solutions was employed as the electrolyte, the CV data acquired in conjunction with exposure to an ultraviolet (UV) light-emitting diode (LED) were almost identical and exhibited little hysteresis. This lack of any effect of the electrolyte on the CV plots indicates a Nernst-like shift in the band-edge potential, meaning that the band edges were always pinned at a constant potential relative to a reversible hydrogen electrode (RHE) regardless of pH.^20^ However, the CV data acquired in the neutral unbuffered electrolyte (0.1 M Na_2_SO_4_) exhibited a unique hysteresis effect, especially at negative potentials in the vicinity of the onset potential. The unique hysteresis discussed herein is evident in the magnified CV data (Fig. 1b). During the sweep from negative to positive potential, the anodic photocurrent initially increased by approximately −0.2 V_RHE_ but then rapidly plateaued. The photocurrent was again increased upon applying potentials more positive than approximately 0 V_RHE_. In the case of the backward scan from positive to negative potential, the apparent onset potential was approximately 0 V_RHE_. Hysteresis in a CV plot depending on the potential scan direction is typically attributed to anodic (photo)currents derived from oxidation of the (photo)electrode materials themselves rather than to oxygen evolution,^21–23^ or to temporal cathodic photoresponse originating from reduction of adsorbates (such as photo-oxidized products like oxygen).^24^ However, these effects cannot explain the different CV data acquired using the Nb:STO photoanode in KPi and Na_2_SO_4_ electrolytes having identical pH values. This unique hysteresis does not appear to be associated with a specific interaction with cation species or a specific adsorption of anion species on the semiconductor surface (Fig. S1 and S2†).^25^

To elucidate the cause of these variations in PEC characteristics with changes in the electrolyte, Mott–Schottky plots were acquired in association with a cyclic potential scan with and without light exposure (Fig. 1c). Details of the impedance assessments are provided in Fig. S3–S7.^26–31^† In the absence of light, the Nb:STO electrode generated almost identical Mott–Schottky plots for all electrolytes. Hence, the flat-band potential was unchanged and n-type semiconductivity with the same carrier density was present in each case, without hysteresis depending on the scan direction. These observations are in agreement with a Nernst-like shift in the band-edge potentials. In trials during which the Nb:STO photoanode was immersed in 0.1 M NaOH or 1 M KPi and exposed to UV light, the Mott–Schottky plots were almost unchanged compared with the plots acquired in the absence of light. That is, using either a highly alkaline or a neutral buffered solution as the electrolyte, the flat-band potential associated with the photoanode was unaffected by the scan direction or by irradiation. These observations are in agreement with the traditional interpretations previously published in the literature.^2^ Only the Mott–Schottky plot acquired with UV irradiation in 0.1 M Na_2_SO_4_ exhibited the unique hysteresis effect. During the potential scan in the negative to positive direction, the flat-band potential was initially observed at approximately −0.3 V_RHE_ but the slope rapidly became zero. The Mott–Schottky plot again showed a positive slope similar to that observed without light upon applying potentials more positive than approximately 0 V_RHE_. In the case of the backward scan from positive to negative potential, the flat-band potential was situated at approximately −0.1 V_RHE_. A significant positive shift of the flat-band potential (indicating band-edge unpinning) was observed only when the electrode potential of the Nb:STO in 0.1 M Na_2_SO_4_ was cathodically swept under UV irradiation. The plateau region in the Mott–Schottky plot, over which the depletion layer capacitance was independent of the applied potential, has been ascribed to Fermi level pinning.^32–34^ In the field of photoelectrochemistry, this phenomenon was originally defined as occurring when the height of the Schottky-like barrier formed at the depletion layer is essentially independent of the electrochemical equilibrium potential of the electrolyte.^35,36^ This is the original definition of Fermi level pinning at a semiconductor/metal interface. Previous publications related to PEC water splitting have reported that, in such cases, the photoanode will suffer from a positive onset potential. Fermi level pinning has been universally considered to be associated with the characteristics of the semiconductor material used. Specifically, the Fermi level of the semiconductor is thought to be pinned at some defect or interfacial state.^32–36^ As another possible explanation, Mott–Schottky analyses in conjunction with continuous exposure to light in previous studies have proposed that the band edges of photoanodes can be displaced to more positive potentials under illumination from their positions in darkness because photogenerated holes are trapped in surface states or accumulated below the surface due to a low PEC reaction rate.^37^ However, these traditional interpretations that ascribes the unique phenomenon to irregular electronic structures in the semiconductor material, such as defects or charge accumulation, cannot fully explain the present unique behaviour. Here, we propose the following reasonable hypothesis. The oxygen evolution reaction in a neutral or acidic medium is accompanied by the release of protons. Consequently, the electrolyte pH in the vicinity of the (photo)anode surface may have decreased as the (photo)anode promoted the oxygen evolution reaction. This local pH gradient would not be expected to form in a highly alkaline or buffered electrolyte.^12^ Neutral unbuffered electrolytes could not mitigate localized decreases in pH, leading to a shift in the band edge to a more positive potential.

### Hydrodynamic voltammetry measurements

**Fig. 2:**
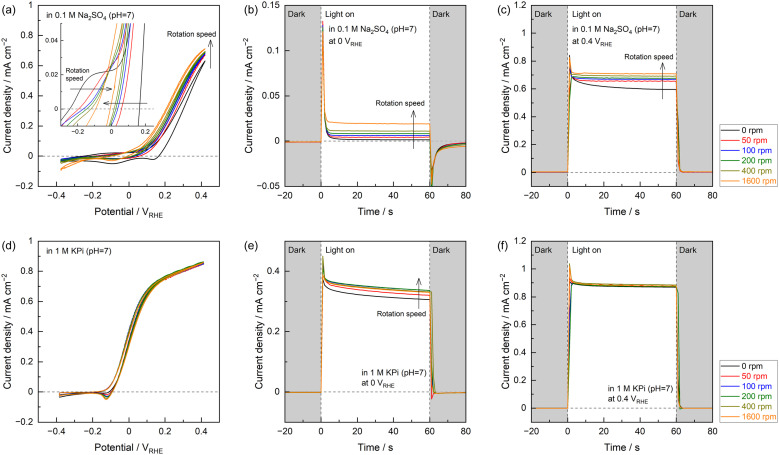
(a) CV data and current–time curves acquired at applied potentials of (b) 0 and (c) 0.4 V_RHE_ with a rotating Nb:STO disk electrode at various rotation rates in a 0.1 M Na_2_SO_4_ electrolyte and (d–f) the same plots obtained in a 1 M KPi electrolyte. A 365 nm LED light source was used.

The contributions of the buffering effect of the electrolyte and of reactant diffusion to PEC performance were evaluated in more detail using a rotating Nb:STO single-crystal disk electrode, as summarized in Fig. 2. The details of the experimental setup are provided in Fig. S8 and S9.† CV curves obtained using the Nb:STO in an unbuffered Na_2_SO_4_ electrolyte under UV light demonstrated a significant effect of the electrode rotation rate over the entire potential region (Fig. 2a). At positive potentials in the CV curves, an increase in the rotation rate increased the anodic photocurrent. At negative potentials around the onset potential, the degree of hysteresis in the CV data with changes in the scan direction was reduced at higher rotation rates. As a consequence, the overall CV plots were gradually shifted in the negative potential direction as the rotation rate was increased. Interestingly, the CVs acquired using the KPi electrolyte were completely unaffected by the electrode rotation rate (Fig. 2d). The evident variation in the photocurrent with changes in the rotation rate confirmed that diffusion processes in the electrolyte governed the PEC reaction to some extent. This effect of diffusion was observed only in the case of the unbuffered electrolyte containing Na_2_SO_4_ and was not apparent when the buffered electrolyte was employed.

The present experimental results also suggest that oxidation of the photoanode surface competed with the oxygen evolution reaction, as a phenomenon other than the diffusion. Using either the Na_2_SO_4_ or KPi electrolytes, the photoanode generated a stable, steady photocurrent at relatively positive potentials with rotation of the electrode (Fig. 2c and f). However, at relatively negative potentials around the onset potential, the photocurrent obtained in the Na_2_SO_4_ electrolyte rapidly decayed over a span of several seconds immediately following the initiation of light exposure (Fig. 2b). Conversely, the photocurrent gradually decreased over time in trials with the KPi electrolyte even when the electrode was rotated (Fig. 2e). These differences in photocurrent stability depending on the electrode potential imply that the photocurrent, especially at negative potentials around the onset potential, was partly a result of oxidation of the photoanode in all electrolytes. Nevertheless, the photoanode in the Na_2_SO_4_ electrolyte was capable of generating a small but steady photocurrent even at negative potentials at which the CV exhibited hysteresis depending on the scan direction (Fig. 2b). It is apparent that not all the photocurrent that appeared in this potential region was associated with oxidation of the semiconductor photoanode. The oxygen evolution reaction also contributed to the photocurrent to some extent.

**Fig. 3:**
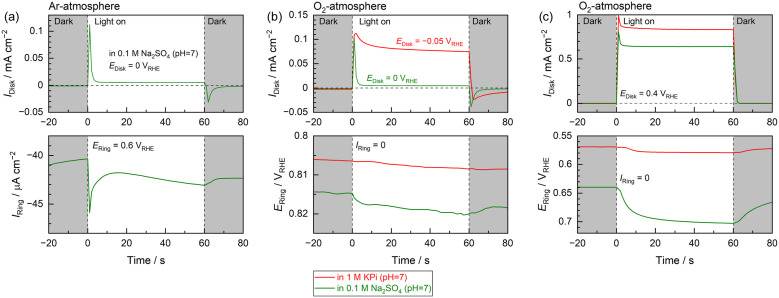
(a) The disk photocurrent and simultaneously recorded ring current obtained from a rotating Pt ring-Nb:STO single-crystal disk electrode under Ar as functions of time. During this trial, a negative potential in the vicinity of the onset (0 V_RHE_) and a relatively positive potential at which the oxygen reduction reaction was able to proceed (0.6 V_RHE_)^39^ were applied to the Nb:STO disk and Pt ring electrodes, respectively, in a 0.1 M Na_2_SO_4_ solution. Changes over time in the disk photocurrent and ring rest potentials as determined under O_2_ by applying (b) negative potentials in the vicinity of the onset potential or (c) a positive potential of 0.4 V_RHE_ to the Nb:STO disk electrode. For the experiments in (b), when the neutral buffered electrolyte (1 M KPi) was employed, an appropriately negative potential was applied to the Nb:STO disk so that the initial photocurrent value occurring 1 s after the initiation of light exposure was similar to that observed when using the Na_2_SO_4_ electrolyte. During these experiments, the RRDE was rotated at 100 rpm and a 365 nm LED light source was used.

Assessing the hypotheses discussed thus far requires evidence of several effects. The first of these is the minute photocurrent generated by the Nb:STO photoanode in the Na_2_SO_4_ electrolyte at negative potentials in the vicinity of the onset potential, which appears to originate from oxygen evolution. The second is the ability of the electrolyte buffering effect to modify changes in the local pH during PEC oxygen evolution. These two phenomena were further investigated using a rotating Pt ring-Nb:STO single-crystal disk electrode (Fig. 3).

The anodic photocurrent generated by the Nb:STO disk photoelectrode in response to UV irradiation and the simultaneously recorded current generated by the Pt ring electrode are plotted in Fig. 3a. The Pt ring electrode generated a cathodic current in response to the anodic photocurrent produced by the Nb:STO disk under UV irradiation. It should be noted that this trial was conducted under Ar and that water-soluble chemicals related to cations in the photoanode (such as Nb, Sr and Ti) were not electrochemically reduced in this potential region (see the ESI† for a more detailed discussion).^38^ Hence, the only chemical species that could be cathodically detected by the Pt ring in the present experiments was the O_2_ evolved at the disk electrode.^39^ The results presented in Fig. 3a confirm that the Nb:STO was capable of producing oxygen even in conjunction with a minute anodic photocurrent by applying a negative potential close to the onset potential in an unbuffered electrolyte. From the rough estimation considering the O_2_ collection efficiencies at the ring electrode (Fig. S10–S13†),^40–42^ it appears that a major portion of the photocurrent obtained several seconds after the initiation of light exposure could be attributed to the oxygen evolution reaction.

The Pt ring electrode rest potential was monitored over time during the PEC oxygen evolution reaction using the Nb:STO disk photoanode, with the results summarized in Fig. 3b and c. Because these measurements were conducted while bubbling O_2_ through the electrolyte, the liquid medium was saturated with O_2_. For this reason, the activity of O_2_ dissolved in the electrolyte would have been constant even in the case that O_2_ was evolved at the disk electrode. The generation of protons in conjunction with the oxygen evolution reaction was anticipated to shift the potential of the Pt ring electrode in the positive direction, reflecting a decrease in pH. Consequently, the Pt ring electrode could detect temporal changes in pH in the vicinity of the disk electrode.^42–44^ Even though the stable photocurrent generated by the Nb:STO disk at negative potentials in the vicinity of the onset potential was very low in the unbuffered electrolyte (0.1 M Na_2_SO_4_), the Pt ring electrode potential was obviously shifted in the positive direction (Fig. 3b). When the neutral buffered electrolyte (1 M KPi) was employed, even though the total charge measured during the PEC reaction in the KPi electrolyte was significantly larger than that observed using the Na_2_SO_4_ solution, the potential associated with the Pt ring in the buffered electrolyte was only minimally shifted. This effect was more evident in the case that a greater reaction rate was obtained by applying more positive potentials to the Nb:STO disk (Fig. 3c). The larger anodic photocurrent generated in the Na_2_SO_4_ electrolyte resulted in a greater pH decrease in the vicinity of the Nb:STO disk compared with that observed at a negative applied potential, as shown in Fig. 3b. The pH variation induced by the PEC reaction was greatly reduced in the KPi electrolyte despite the large anodic photocurrent. These data establish that the pH in the vicinity of the photoanode was decreased as the PEC oxygen evolution reaction proceeded even in conjunction with forced convection of electrode rotation. It is also apparent that localized changes in pH were drastically suppressed when using the buffered electrolyte.

### Buffer concentrations

**Fig. 4:**
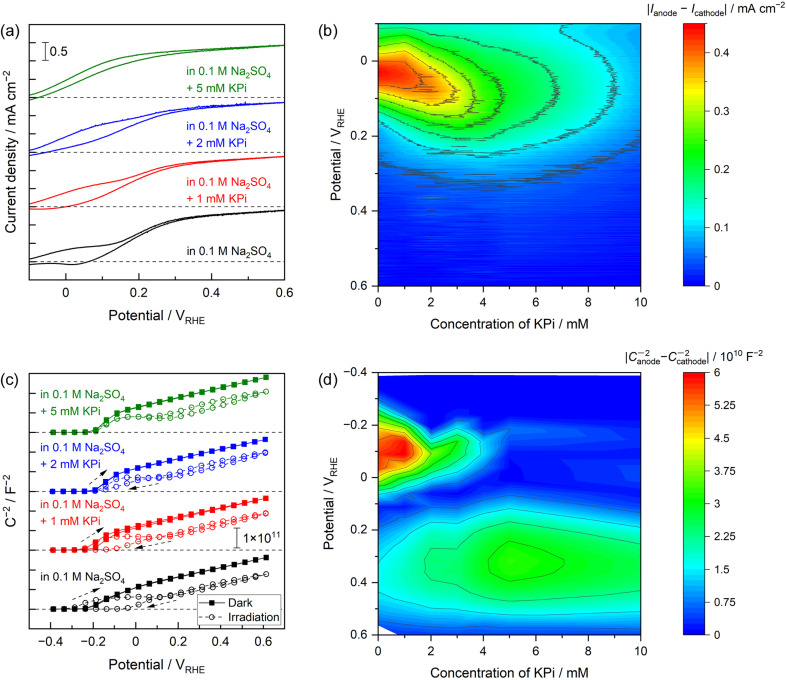
(a) CV data obtained using the Nb:STO photoanode in 0.1 M Na_2_SO_4_ electrolytes containing various concentrations of KPi with illumination by a 365 nm LED and (b) photocurrent hysteresis as functions of the electrode potential and KPi concentration. (c) Mott–Schottky plots acquired with and without UV irradiation and (d) the degree of hysteresis in the plots acquired with light exposure as functions of the electrode potential and KPi concentration. In these trials, low concentrations of KPi on the order of several millimolar were added to an aqueous 0.1 M Na_2_SO_4_ solution as a supporting electrolyte. Arrows indicate the potential scan direction.

The effects of the buffer concentration on the PEC performance of the Nb:STO photoanode were further examined, with the results presented in Fig. 4. The plateau photocurrent values recorded in the vicinity of the onset potential during the anodic potential scan were found to gradually increase. In addition, the apparent onset potentials observed during the backward cathodic scan were shifted to more negative values (Fig. 4a). That is, as the KPi concentration was increased, the CV curves exhibited reduced hysteresis and thus adopted the more ideal shapes observed in trials using the highly alkaline electrolyte. The absolute values of the differences between photocurrents observed during the anodic and cathodic scans are plotted as functions of the KPi concentration and applied potential in Fig. 4b. These values indicate the degree of hysteresis in the CV data, with the red plots showing significant hysteresis. In the absence of KPi or at low concentrations of up to approximately 3 mM, the colour map shows a large degree of hysteresis at negative potentials around 0 V_RHE_. However, after increasing the KPi concentration to approximately 5–10 mM, the hysteresis was greatly decreased. The effect of the KPi concentration on the Mott–Schottky plots is also evident in Fig. 4c. In the absence of light, the electrolyte composition had a minimal effect on the plot shape. Conversely, under UV radiation, increasing the KPi concentration partly suppressed the hysteresis seen in the Mott–Schottky plots acquired with light irradiation. The absolute values of the differences between the *C*^−2^ values acquired from Mott–Schottky plots generated with anodic and cathodic scans under UV light are also plotted as functions of the KPi concentration and applied potential in Fig. 4d. A large degree of hysteresis is apparent, especially at negative potentials in the vicinity of −0.1 V_RHE_ for low KPi concentrations (up to approximately 3 mM). In contrast, higher concentrations of 5–10 mM provided little hysteresis. This relationship between the KPi concentration and hysteresis is consistent with the trends exhibited by the CV data. The most important finding here is that very low KPi concentrations of only 5–10 mM reduced the extent of hysteresis in the CV data and so limited undesirable band-edge unpinning. This concentration range could be insufficient to provide the ionic conductivity required from a supporting electrolyte during the electrochemical process but did show high buffering capacity compared with an unbuffered electrolyte such as a Na_2_SO_4_ solution (Fig. S14†). Thus, it can be concluded that the buffering effects of the electrolyte made an important contribution to band-edge pinning and that an electrolyte with a greater buffering capacity produced a more ideal semiconductor/electrolyte interface even during the PEC oxygen evolution reaction at a neutral pH. However, it should also be noted that the Mott–Schottky plot obtained under UV light in the electrolyte containing 5 mM KPi actually exhibited less hysteresis but still demonstrated a plateau region irrespective of the potential scan direction (Fig. 4c). This finding implies that, in the case that the buffering capacity exceeded that of the neutral unbuffered electrolyte but was still relatively weak, the band edge might not have been completely pinned. This scenario would result in a slight shift of the band edges to positive potentials during the PEC reaction.

Increasing the KPi concentration to 1 M caused the shapes of the current–potential curves obtained using the Nb:STO to gradually improve. In addition, the Mott–Schottky plots acquired with UV irradiation no longer exhibited a plateau at negative potentials. As such, these plots took on shapes similar to those of plots acquired in highly alkaline solutions and/or dark conditions (Fig. S15†). The improved shapes of the current–potential curves can be explained based on accelerated oxygen evolution kinetics (Fig. S16†). When the electrolyte contained relatively low concentrations of KPi (up to approximately 10 mM), water molecules served as the main reactant to form oxygen due to limitations on the diffusion of protonated buffered species. Meanwhile, an effect of diffusion on the current was no longer observed in more dense buffered solutions containing higher KPi concentrations, suggesting that oxygen evolution involving buffering anions was the dominant reaction. This phenomenon represents reactant switching depending on the buffer concentration, as has been reported to occur in the field of electrocatalytic water splitting.^9^ The disappearance of the plateau region in the Mott–Schottky plots indicates that additional increases in buffering capacity suppressed band-edge unpinning more strongly.

### Bulk pH of unbuffered electrolytes

**Fig. 5:**
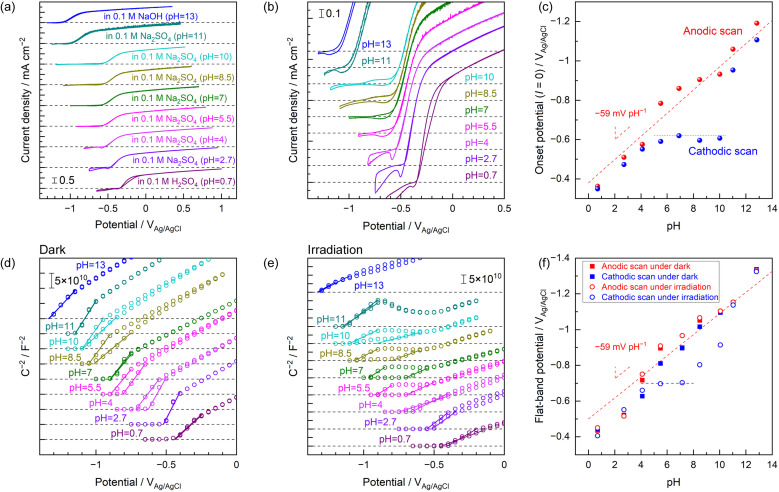
(a) Full-scale and (b) magnified CV data obtained using a Nb:STO photoanode in electrolytes with various pH. (c) Apparent onset potentials observed during anodic or cathodic potential scans as functions of the electrolyte pH. Here, the onset was defined as the electrode potential at which the photocurrent value was equal to zero. Mott–Schottky plots acquired in electrolytes with various pH in conjunction with a cyclic potential scan (d) without and (e) with UV irradiation. (f) Apparent flat-band potentials estimated from the anodic and cathodic scans without and with UV irradiation as functions of the electrolyte pH. The apparent flat-band potentials were estimated from the linear slopes in the vicinity of the onset in the Mott–Schottky plots. These linear fittings are indicated by solid lines in (d and e).

The effects of the bulk pH of the unbuffered electrolyte on the shape of CVs acquired under UV light were assessed, with the results compiled in Fig. 5. Full-scale CVs and magnified views around the onset potentials are presented in Fig. 5a and b, respectively. Hysteresis of the anodic photocurrent depending on the potential scan direction was almost never observed at highly acidic pH values up to approximately 4 or at highly alkaline pH values above 11. The unique hysteresis phenomenon occurring around the onset potentials was evident at near-neutral pH values ranging from approximately 5 to 10. The effects of pH on the onset potentials during the forward anodic scan and the backward cathodic scan are summarized in Fig. 5c. The onset potentials observed during the anodic scan were found to be negatively shifted with increases in pH, indicating Nernst-like behaviour. In highly acidic or alkaline media, the onset potentials during the cathodic scan also exhibited this Nernst-like shift. However, the onset potentials observed during the cathodic scan under near-neutral pH conditions were pinned at an almost constant potential of −0.6 V *vs.* Ag/AgCl. The rising shapes of CVs produced at potentials more positive than approximately −0.6 V *vs.* Ag/AgCl within this near-neutral pH range were almost independent of the pH of the bulk electrolyte (Fig. 5a and b). The entire Mott–Schottky plots acquired under dark conditions were negatively shifted in response to increases in pH while maintaining almost the same slope and shape (Fig. 5d). These plots showed very minimal hysteresis with changes in the potential scan direction. In contrast, only the Mott–Schottky plots obtained in the near-neutral unbuffered electrolytes with pH values of approximately 5–10 under UV light demonstrated the unique hysteresis in the vicinity of the onset potential (Fig. 5e), similar to the trend shown by the CVs. Comparisons of the Mott–Schottky plots obtained in the unbuffered electrolytes having various pH values without and with UV irradiation without offset in the *y*-axis direction are included in Fig. S17.† The shapes of Mott–Schottky plots acquired with UV exposure at near-neutral pH values and potentials more positive than approximately −0.5 V *vs.* Ag/AgCl were almost identical regardless of the bulk pH. This result was in contrast to the monotonic shift of the plots with changes in pH under dark conditions. The apparent flat-band potentials determined from the anodic and cathodic scans of the Mott–Schottky plots acquired without and with UV exposure are shown as functions of the electrolyte pH in Fig. 5f. Data acquired in the absence of light or from anodic scans under UV irradiation exhibited a Nernst-like shift in the apparent flat-band potentials. Even the Mott–Schottky plots produced using cathodic scans under UV light showed this ideal behaviour in highly acidic or alkaline solutions. The flat-band potentials were positively shifted only during the backward cathodic scans in the near-neutral unbuffered electrolytes under UV irradiation. These results demonstrate that the unique hysteresis seen in the vicinity of the onset potentials in the CV data acquired in near-neutral electrolytes under UV irradiation (Fig. 5a and b) were related to band-edge unpinning rather than variations in the oxygen evolution reaction kinetics in the electrolyte.^9^ It should be noted here that the apparent flat-band potentials observed during the cathodic scans under UV irradiation were fixed at approximately −0.7 V *vs.* Ag/AgCl at pH values up to approximately 8 but showed a slow gradual shift in the negative direction for pH values of 8–10. Interestingly, when weakly alkaline conditions with pH values of 8–10 were employed, the Mott–Schottky plots generated from cathodic scans under UV light exhibited small plateaus at relatively negative potentials around the onset (Fig. S18†). The *C*^−2^ values within this plateau region during the cathodic scans gradually increased in response to increases in pH. The Mott–Schottky plots acquired in the pH 11 electrolyte eventually exhibited less hysteresis. However, unusual variation of the slope values was seen at potentials ranging from −0.9 to −0.6 V *vs.* Ag/AgCl irrespective of the potential scan direction. This trend is similar to that induced by varying the KPi concentration, especially in the low concentration range, as shown in Fig. 4. Increasing the pH within the weakly alkaline region gradually moved the semiconductor/electrolyte interface closer to the ideal band-edge pinning state observed in highly alkaline solutions. These results also demonstrate that even the pH 11 electrolyte produced an undesirable positive shift of the band edges under UV irradiation, despite the absence of the unique hysteresis effect in the CV. Indeed, this gradual variation in the apparent flat-band potentials in response to UV light at pH values above 8 was also consistent with increases in buffer strength of the Na_2_SO_4_ solution in this pH region with increases in pH (Fig. S14†). Based on these data, we propose that the band edges of the photoanode were pinned at potentials reflecting the bulk electrolyte pH irrespective of the buffering effect of the electrolyte, exposure to light or the pH value, so long as oxygen evolution did not occur. This represents ideal Nernst-like behaviour. However, when the oxygen evolution reaction was initiated by illuminating the photoanode in the near-neutral unbuffered electrolyte, band-edge pinning was no longer maintained due to the local pH gradient generated in the vicinity of the semiconductor/electrolyte interface. The extent of the positive shift in the band-edge potentials (that is, the extent to which the pH in the vicinity of the photoanode decreased) reflected the buffering capacity of the electrolyte. It should be noted here that non-Nernstian relationship between the pH and the flat-band potential has sometimes been explained by the thermodynamic unbalance between the proton adsorption and desorption processes (*e.g.*, unequal reaction free energies for these two processes).^7^ However, the unique band-edge unpinning in the present study were observed only during the cathodic scan Mott–Schottky plots under UV irradiation in near-neutral unbuffered electrolytes. Otherwise, the flat-band potentials of Nb:STO followed almost ideal Nernst-like behaviour (that is, approximately −59 mV pH^−1^ of slopes in Fig. 5f). Therefore, it should be reasonable to conclude that the present unique phenomena were derived from the standard equilibrium of surface protonation/deprotonation depending on the local pH rather than any changes in mechanisms during these surface protonation/deprotonation processes.

### Interacting contributions of semiconductor characteristics and physicochemical phenomena in electrolytes

**Fig. 6:**
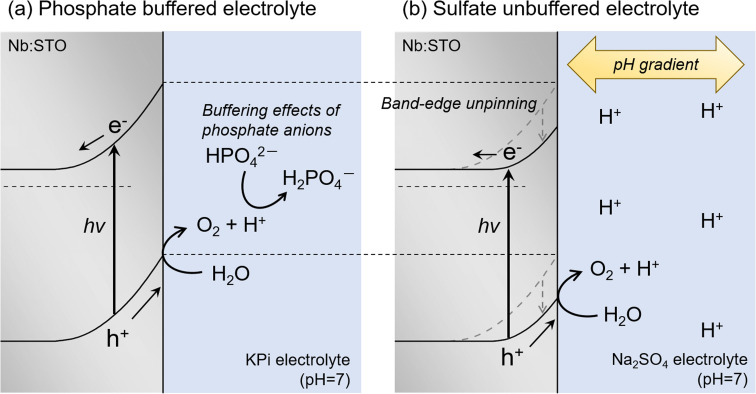
Diagrams explaining the effects of (a) a neutral buffered electrolyte and (b) a neutral unbuffered electrolyte on the band diagram for the photoanode/electrolyte interface. Note that the scales do not necessarily reflect the actual size relationships.

Fig. 6 provides diagrams showing the manner in which the energy band diagram at the semiconductor/electrolyte interface was modified by electrolyte buffering. In the case that a neutral buffered electrolyte such as a KPi solution was employed, protons released during water oxidation were readily consumed by the protonation of phosphate anions,^9^ such that pH variations in the vicinity of the photoanode surface were suppressed (Fig. 6a). As a consequence, the photoanode in the neutral buffered electrolyte exhibited the ideal band-edge potential reflecting the pH of the bulk electrolyte regardless of the presence or absence of light, as was also the case when using a highly alkaline electrolyte. The protons produced during water oxidation in a neutral unbuffered electrolyte such as Na_2_SO_4_ greatly lowered the pH close to the photoanode surface compared with that in the bulk electrolyte (Fig. 6b). This localized pH gradient positively shifted the band-edge potentials. This phenomenon would appear to correspond to Fermi level pinning. It should be emphasized that the present observations are attributed to physicochemical processes occurring in the electrolyte rather than to electronic defects in the semiconductor. The latter is often assumed in the literature.^32–36^ Indeed, the hysteresis behaviours in the CV curves during the PEC reaction involving the proton-coupled electron transfer were observed regardless of the choice of metal oxide photoanodes (Fig. S19 and S20†), supporting that these phenomena should be mainly governed by the transient physicochemical phenomena inside the electrolyte.

The important point here is that the band edges did not continue to shift positively with ongoing positive shifts of the applied potential (*i.e.*, with increases in the PEC reaction rate). Rather, applying positive potentials above a specific level recovered the positive slopes of the Mott–Schottky plots as well as the anodic photocurrent. These findings can be understood by considering that the CVs acquired in the neutral unbuffered electrolyte showed the unique hysteresis effect even in the presence of forced convection with vigorous stirring or electrode rotation but that increasing the rotation rate of the RRDE to produce a thinner diffusion layer certainly reduced hysteresis in the CVs. It appears that the band-edge potentials were predominantly governed by the pH in proximity to the photoanode surface (such as Helmholtz layer) rather than changes in the overall pH gradient across the thick diffusion layer. The total amount of protons produced in the vicinity of the photoanode surface increased in association with progress of the PEC oxygen evolution reaction. However, because the thickness of the diffusion layer also gradually increased, the proton concentration in the immediate vicinity of the Helmholtz layer was unable to increase above a certain level. As a consequence, the band edges did not shift in the positive direction beyond a certain point. It can be considered that, when the applied potentials begin to contribute to the growth of the depletion layer inside the semiconductor at potential region more positive than this threshold, the photocurrent should be mostly governed by the increased built-in potential resulting in the similar rising shapes of CVs.

## Conclusions

The present study suggests that the use of an optimal electrolyte is an important aspect of achieving superior PEC performance for a given photoelectrode material. As an example, a buffered electrolyte should be used in the case that PEC water splitting is to be performed at a near-neutral pH. However, the fundamental principle underlying this assertion is different from the conventional viewpoint. For instance, in the case that a photoanode shows a more positive onset potential compared with the reported conduction band minimum or the flat-band potential determined from Mott–Schottky plots acquired under dark conditions, this material is typically regarded as less active for PEC oxygen evolution. In the standard view, this poor PEC performance is ascribed to the presence of electronic defects in the semiconductor. Alternatively, low PEC performance can be attributed to the electrolyte. In such cases, the water splitting reaction kinetics in a neutral unbuffered medium are considered to be sluggish compared with those in highly alkaline or neutral buffered solutions. Nernstian voltage losses in the electrolytes can also adversely affect the PEC performance. All explanations are premised on the ideal band diagram at the semiconductor/electrolyte interface, meaning that the band edges are pinned at appropriate potentials. Rather, the present study instead proposes another possibility that the electronic band structure at the semiconductor/electrolyte interface can be affected as a consequence of a transient physicochemical phenomenon in the electrolyte (*i.e.*, the formation of a localized pH gradient) resulting from the PEC oxygen evolution reaction. This new insight suggests that the overall PEC performance may be determined by interacting contributions of both the semiconductor material and the electrolyte rather than solely by conventional physics or electrochemistry. This effect should be taken into account when interpreting the results of PEC trials.

## Author contributions

Y. Kageshima conceptualised the research, established the methodology, performed all the (photo)electrochemical measurements and wrote a draft of the manuscript. H. Kumagai contributed to the interpretation of the experimental results and reviewing and editing of the manuscript. K. Teshima and K. Domen supervised the research. H. Nishikiori supervised the research and contributed to reviewing and editing of the manuscript. All authors contributed to the research, discussed the results and approved the final version of the manuscript.

## Conflicts of interest

There are no conflicts to declare.

## Supplementary Material

SC-OLF-D5SC01816A-s001

## Data Availability

The data supporting the findings of this study are available within the paper and the ESI.† All relevant data are available from the corresponding authors on request.
