# Supplementary material for: Neutral buffered electrolytes guarantee ideal band-edge pinning for semiconductor photoanodes
Source: Chem Sci. 2025 Jul 10;16(31):14088–97. doi: 10.1039/d5sc01816a (PMC12257357; doi:10.1039/d5sc01816a)
Supplement: SC-016-D5SC01816A-s001 [file SC-016-D5SC01816A-s001.pdf]

Supplementary Information (SI) for Chemical Science.
This journal is © The Royal Society of Chemistry 2025

Supplementary Information

Neutral buffered electrolytes guarantee ideal band-edge pinning for semiconductor photoanodes

Yosuke Kageshima,*[ab] Hiromu Kumagai,[c] Katsuya Teshima,[ab] Kazunari Domen [bd]
and Hiromasa Nishikiori *[ab]

a Department of Materials Chemistry, Faculty of Engineering, Shinshu University, 4-17-1 Wakasato, Nagano 380-8553, Japan.

b Research Initiative for Supra-Materials (RISM), Shinshu University, 4-17-1 Wakasato, Nagano 380-8553, Japan.

c Research Center for Advanced Science and Technology, The University of Tokyo, 4-6-1 Komaba, Meguro-ku, Tokyo 153-8904, Japan.

d Office of University Professors, The University of Tokyo, 7-3-1 Hongo, Bunkyo-ku, Tokyo 113-8656, Japan.

Experimental Section

Electrode preparation: A commercially-available Nb-doped $SrTiO_3$ (Nb:STO) single crystal having a [100] surface orientation (Shinkosha, Kanagawa, Japan; 0.01 wt% Nb, 0.5 mm thick) was used as the photoanode. Wiring was attached to the back side of a 10 × 10 mm wafer using indium soldering and the back and sides of the crystal were subsequently covered with epoxy resin. The photoanode produced in this manner was employed during PEC trials in which the photoanode surface was placed perpendicular to the cell bottom. The results presented in Figures 1, 4 and 5 were acquired in this way. STO was employed because this material is known to exhibit high efficiency during photocatalytic overall water splitting[1] and PEC oxygen evolution.[2] In addition, because surface roughness can complicate the interpretation of impedance data,[3] a smooth, flat single-crystal wafer was used.

A Nb:STO single crystal disk with a diameter of 4 mm was used for hydrodynamic voltammetry experiments in conjunction with a rotating ring disk electrode (RRDE). In preparation for these analyses, the Nb:STO disk was attached to a commercially-available disk replaceable electrode (BAS, Tokyo, Japan). The details of this process are described in the following section. The results presented in Figures 2 and 3 were obtained using this Nb:STO RRDE.

Particulate TiO_2 or WO_3 photoanodes were prepared as follows. For fabrication of particulate TiO_2 photoanodes, a fluorine-doped thin oxide-coated glass (FTO/glass) substrate was sequentially cleaned with acetone, ethanol, and water and then coated with a TiO_2 thin film by the sol–gel method. A paste containing commercially available TiO_2 particles (Aerosil AEROXIDE, P25) was prepared using a planetary centrifugal mixer (Shashin Kagaku, Kyoto, Japan; Kakuhunter, SK-300SII) and then deposited using a squeegee method onto the FTO/glass substrate coated with a TiO_2 sol–gel thin film. The resultant specimen was calcined at 723 K for 30 min.[4] For fabrication of particulate WO_3 photoanodes, a paste containing commercial WO_3 particles was deposited on the bare FTO/glass by a squeegee method, and the resultant specimen was calcined at 773 K for 30 min.

(Photo)electrochemical assessments: An aqueous solution containing 0.1 M NaOH was used as a highly alkaline electrolyte while neutral aqueous solutions containing 0.1 M Na_2SO_4 or 1 M potassium phosphate (KPi; 0.5 M K_2HPO_4/0.5 M KH_2PO_4) were employed as unbuffered or buffered electrolytes, respectively. STO-based photoanodes are known to show high levels of photoelectrochemical (PEC) performance in highly alkaline electrolytes such as 0.1 M NaOH.[2] In other work, 1 M KPi or 0.1 M Na_2SO_4 solutions have typically been used as buffered or unbuffered neutral electrolytes, respectively.[5] The pH values of the Na_2SO_4 and KPi electrolytes were adjusted

to 7 using NaOH or KOH, respectively. Note that the pH values of the electrolytes based on Na_2SO_4 and various concentrations of KPi (see Figure 4) were adjusted to 7 using only NaOH, and that Figures 4b and 4d were constructed from the results acquired using 0.1 M Na_2SO_4 without KPi and the electrolyte containing 1, 2, 3, 4, 5, or 10 mM of KPi (i.e., total seven data points). Each of these electrolytes was purged with Ar unless otherwise noted. During a typical PEC experiment, the electrolyte was vigorously stirred using a magnetic bar (see Figures 1a, 4a, 4b, 5a and 5b), while Mott-Schottky plots (Figures 1c, 4c, 4d, 5d and 5e) were acquired without forced convection.

A typical three-electrode configuration was used during the (photo)electrochemical analyses, with a Ag/AgCl electrode in a saturated aqueous KCl solution and a Pt coil as the reference and counter electrodes, respectively. A commercial potentiostat (Meiden Hokuto, Tokyo, Japan; HZ-7000) equipped with a frequency response analyzer or a bipotentiostat apparatus were utilized during the impedance and RRDE trials, respectively. During cyclic voltammetry (CV) assessments, the potentials of working electrodes were changed at a scan rate of 10 mV s^{-1} and, during the impedance measurements, a potential amplitude of 10 mV was applied. The impedance values obtained at a frequency of 5 kHz were converted to Mott-Schottky plots. During the RRDE trials, a commercially-available electrode rotator system (BAS, RRDE-3A) was used. The experimental setup is explained in detail in the following section. Note that there have been several studies to date in which the PEC reaction has been evaluated using an RRDE.[6–9] However, such studies have typically involved relatively simple redox reactions as model PEC processes. Thus, this technique has rarely been applied to the assessment of PEC water splitting. This lack of research might be related to the challenges associated with the evaluation of reactions involving the formation of bubbles (such as those of hydrogen and oxygen) using the RRDE technique.[10,11] Even so, hydrodynamic voltammetry is a powerful means of detecting relatively small amounts of products in the vicinity of the (photo)electrode on time scales ranging from several seconds to a few tens of seconds, and so was employed in the present study.

During typical PEC and impedance experiments (Figures 1, 4 and 5), 365 nm monochromatic light from a light-emitting diode (LED; Asahi Spectra, Tokyo, Japan; CL) equipped with a rod lens and operating at 100% output power was employed. An ultraviolet (UV) LED light source equipped with a wide-angle lens and operating at 50% output power was employed during the RRDE analyses.

Results and Discussion

Effects of cation or anion species in the unbuffered electrolyte on the shapes of current-potential curves: The CV data acquired using a Nb:STO photoanode in Na_2SO_4 or K_2SO_4 electrolytes are compiled in Figure S1. A unique hysteresis phenomenon in the vicinity of the onset potential was observed regardless of the specific cation species, suggesting that the anion rather than the cation had an effect.

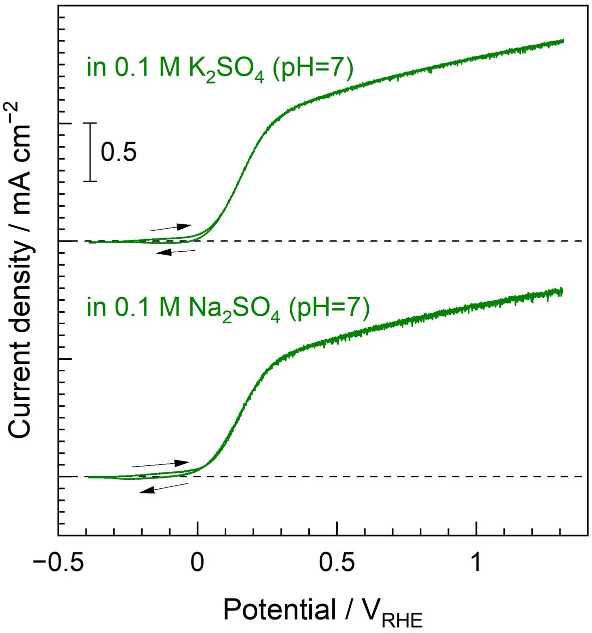

Figure S1. CV data obtained using a Nb:STO single crystal photoanode in aqueous electrolytes containing 0.1 M Na_2SO_4 or K_2SO_4 adjusted to pH = 7. The electrolytes were purged with Ar and vigorously stirred. Light source: 365 nm LED equipped with a rod lens (100% output power). Scan rate: 10 mV s^{-1}.

It has been reported that specific anions such as sulfate and phosphate will rapidly adsorb onto a TiO_2 surface even at millimolar concentrations and consequently reduce the photocatalysis rate.[12] In contrast, perchlorate anions rarely adsorb on the photocatalyst surface.[12] The effect of this adsorption was investigated by acquiring CV data using a Nb:STO photoanode in unbuffered electrolytes containing SO_4^{2-} or ClO_4^- anions, with the results presented in Figure S2. At a neutral pH, a hysteresis effect was found in the vicinity of the onset potential irrespective of the type of anion (Figure S2a). This hysteresis was found to disappear in highly acidic electrolytes (Figure S2b). Considering that sulfate and perchlorate anions, which were respectively likely and unlikely to adsorb, produced similar CV plots, it appears that this hysteresis can be attributed to the pH of the electrolyte rather than the adsorption of anions on the semiconductor surface.

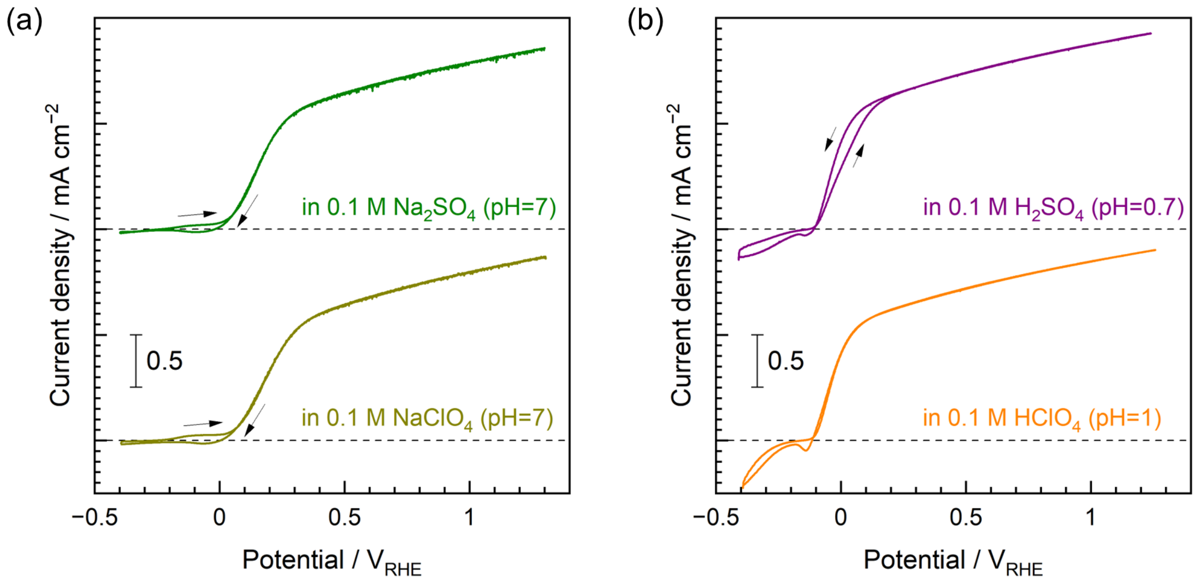

Figure S2. CV data obtained using a Nb:STO single crystal photoanode in (a) neutral and (b) acidic electrolytes containing 0.1 M SO_4^{2-} or 0.1 M ClO_4^-. The electrolytes were purged with Ar and vigorously stirred. Light source: 365 nm LED equipped with a rod lens (100% output power). Scan rate: 10 mV s^{-1}.

Discussion regarding impedance analyses: The optimal frequency required to obtain Mott-Schottky plots was determined based on an assessment of Nyquist and Bode plots as follows. The effects of the electrode potential and of the presence or absence of UV irradiation on the impedance spectra were assessed, with the results presented in Figure S3. Note that these experiments employed a neutral potassium phosphate buffered electrolyte (1 M KPi, pH = 7) without forced convection. The shapes of the impedance spectra, especially in the low-frequency region, were found to be greatly affected by the application of UV light. Consequently, Nyquist plots exhibited smaller arcs when acquired with irradiation for a given applied potential. Irrespective of the presence or absence of irradiation, both the Nyquist and Bode plots showed an effect of the electrode potential over the entire frequency range of 1 Hz to 10 kHz. Hence, the impedance values obtained in this frequency range reflected the characteristics of the photoanode/electrolyte interface.

The effects of irradiation were also examined by comparing the Nyquist and Bode plots acquired at an applied potential of 0 V vs. Ag/AgCl with and without exposure to UV light (Figure S4). Photoexcitation of the Nb:STO only minimally changed the impedance spectra over a frequency range of several kilohertz but significantly altered the shape of plots at lower frequencies. Therefore, the impedance spectra acquired at frequencies below 1 kHz were affected both by the physical processes occurring inside the semiconductor and by the oxygen evolution reaction kinetics at the photoanode surface.

Forced convention via magnetic stirring was found to barely affect the impedance spectra in the absence of light (Figure S5). This result was not unexpected because the use of the Nb:STO photoanode in conjunction with a relatively positive applied potential was known to be incapable of promoting any reactions under dark conditions. In contrast, in the case that the photoanode was exposed to UV light such that the PEC oxygen evolution reaction proceeded at the surface, stirring induced significant noise in the impedance spectra in the low-frequency region below several tens of hertz. This outcome indicates that diffusion processes in the electrolyte could have affected the impedance measurements within this frequency region. The acquisition of Mott-Schottky plots requires the assessment of the effect of the electrode potential on the depletion layer capacitance inside the semiconductor separately from the impedance related to the kinetics of chemical reactions and reactant diffusion. Therefore, the impedance data obtained at frequencies on the order of several kilohertz should be optimal, at least for the present experimental system. For this reason, the Mott-Schottky plots presented in the main manuscript of this study were acquired at a frequency of 5 kHz.

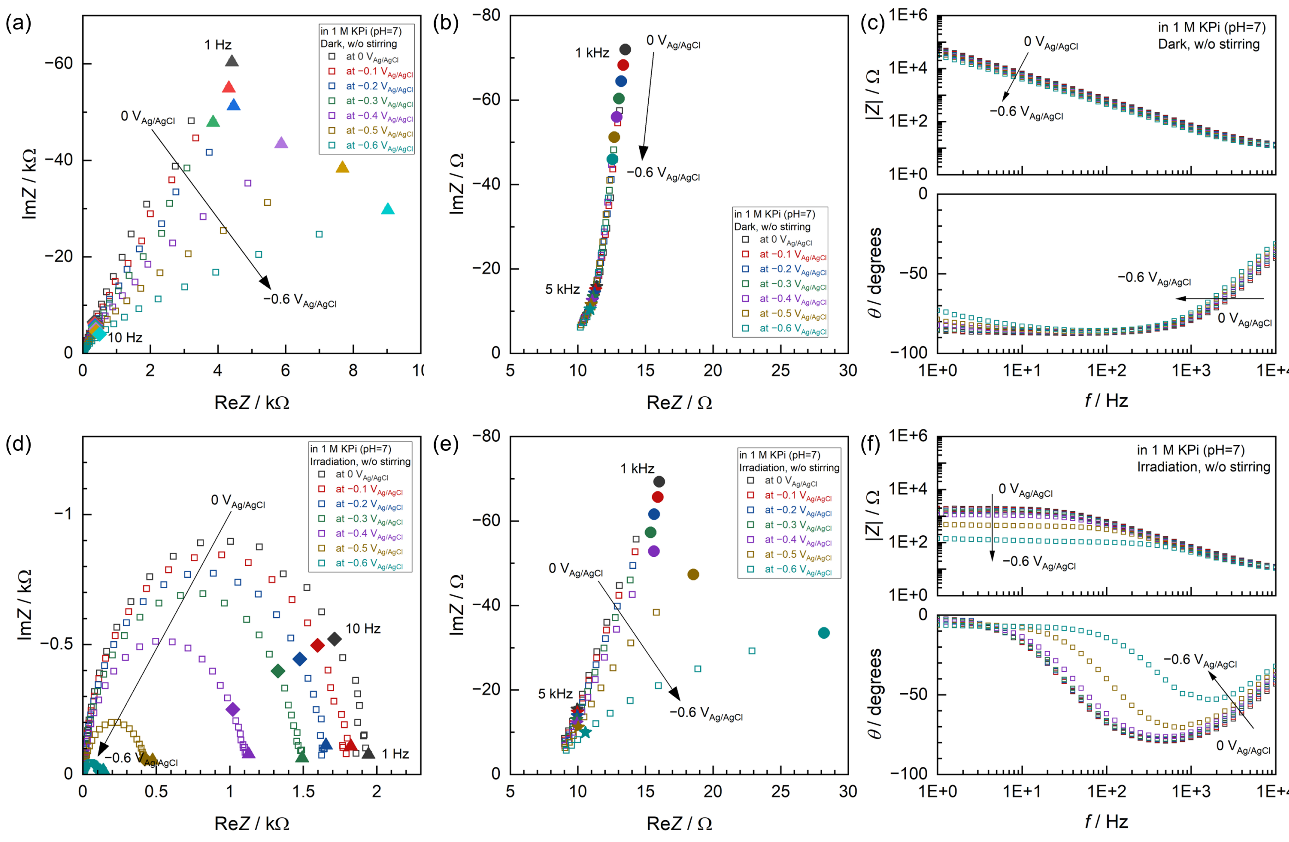

Figure S3. (a, b) Nyquist and (c) Bode plots obtained from a Nb:STO photoanode in conjunction with various applied potentials in the absence of light and (d – f) the same plots acquired but with illumination by a 365 nm LED light source. Data were generated using a neutral buffered electrolyte (1 M KPi, adjusted to pH = 7) without forced convention.

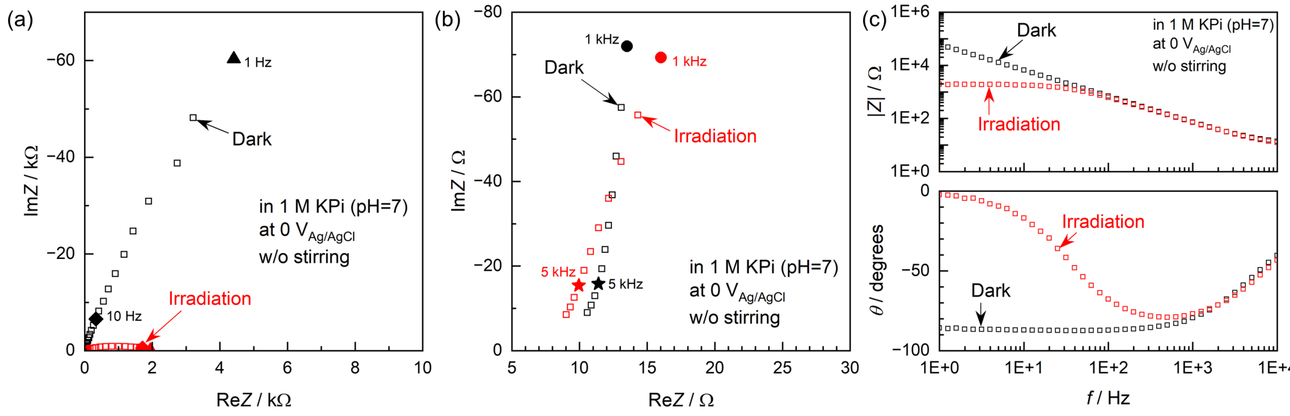

Figure S4. (a, b) Nyquist and (c) Bode plots obtained from a Nb:STO photoanode at an applied potential of 0 V vs. Ag/AgCl in the presence or absence of UV irradiation, employing a neutral buffered electrolyte without forced convention.

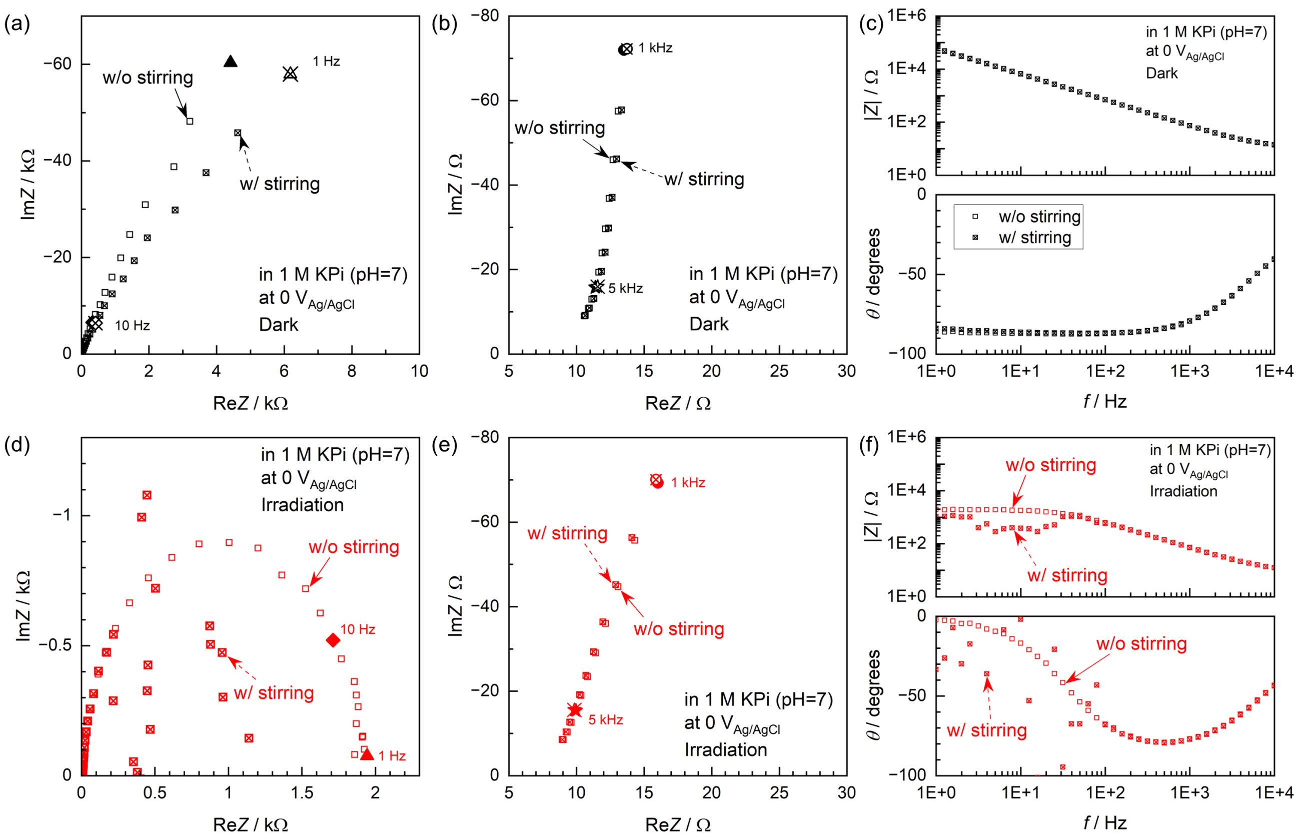

Figure S5. (a, b) Nyquist and (c) Bode plots obtained from a Nb:STO photoanode at an applied potential of 0 V vs. Ag/AgCl without exposure to light with and without forced convection using a magnetic stirring bar and (d – f) the same plots acquired with UV irradiation and a neutral buffered electrolyte.

We also conducted the equivalent circuit fitting for the selected impedance spectra obtained from Nb:STO in the neutral buffered electrolyte under dark condition as summarized in Figure S6. Mott-Schottky plots obtained from the equivalent circuit fitting or from the impedance recorded only at 5 kHz were compared in Figure S7. The similar apparent flat-band potentials (E_{fb}) were obtained regardless of the estimation method of the depletion layer capacitance. Meanwhile, assuming that the dielectric constant of Nb:STO at room temperature is 300,[13] the carrier (donor) density (N_D) values estimated from the former and the latter methods were calculated to be 4.5×10^{18} or 2.0×10^{18} cm^{-3}, respectively. While these values were of similar order, they certainly varied depending on the analysis methods. This is because the accurate fittings for the impedance spectra related to the photoelectrode/electrolyte interface require the constant phase element (CPE) being involved in the equivalent circuit while the Mott-Schottky analyses based on the impedance measured at a single perturbation frequency assume the simple capacitance representing the depletion layer. Nevertheless,

it should be emphasized that the present carrier densities estimated by either method were in relatively good agreement with the reported values (10^{18}~10^{19} cm^{-3} for 0.01 wt% Nb:STO).[14] Therefore, we concluded that the present analysis method (i.e., Mott-Schottky plots acquired at a single frequency of 5 kHz) was sufficiently valid to assess the influence of the electrolyte compositions.

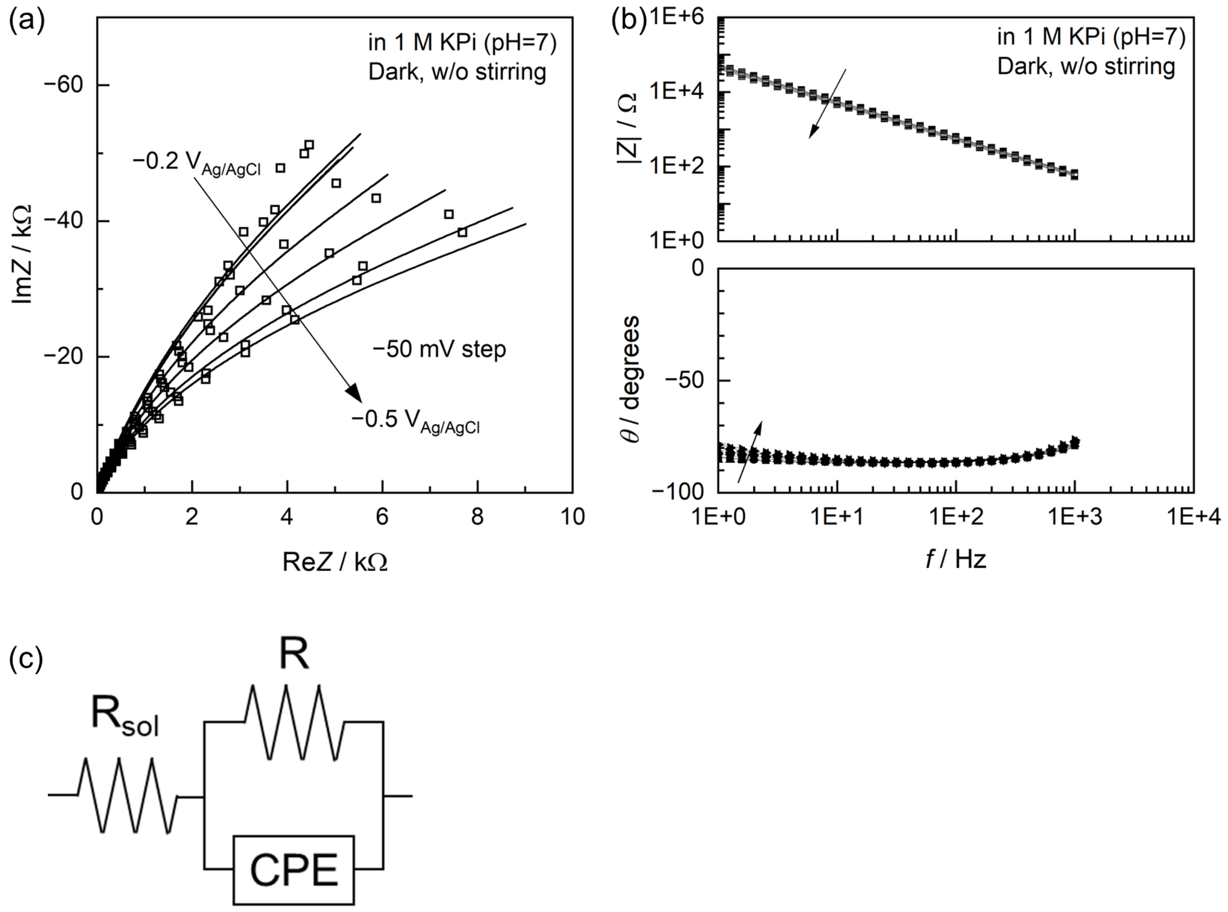

Figure S6. (a) Nyquist and (b) Bode plots obtained from a Nb:STO photoanode in conjunction with various applied potentials in the absence of light. Data were generated using a neutral buffered electrolyte (1 M KPi, adjusted to pH = 7) without vigorous stirring. The fitted curves were overlaid as lines on the measured points. (c) Equivalent circuit employed for the fitting.

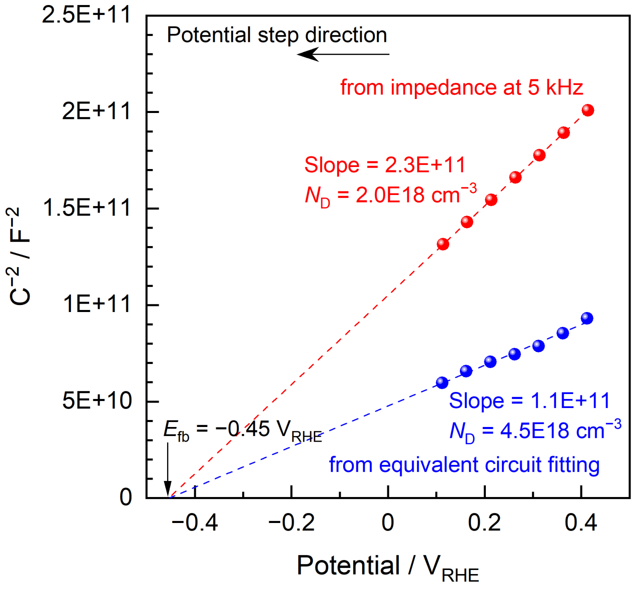

Figure S7. Mott-Schottky plots obtained from the equivalent circuit fitting shown in Figure S6 or from the impedance recorded at a single frequency of 5 kHz.

Note that the slope of Mott-Schottky plots (see Figure 1c in the main manuscript) changed at approximately −0.15 V vs. a reversible hydrogen electrode (RHE). Previously, such inflection points have been attributed to variations in surface morphology on the microscopic scale[15] or to the existence of both shallow and deep donor levels.[3,16] As another possibility, the partial distribution of the applied potential across the Helmholtz layer may cause the nonlinearity in Mott-Schottky plots.[17] It has been theoretically demonstrated that this potential drop within the Helmholtz layer becomes pronounced when the carrier concentration of photoanode is quite high (e.g., 10^{19}~10^{20} cm^{-3}) and/or when the Helmholtz layer capacitance is small. Note that a single-crystal wafer having a planar geometry and a moderate carrier concentration (~10^{18} cm^{-3})[14] was employed as the electrode for these experiments, and that Mott-Schottky plots with similar shapes and the inflection points were obtained irrespective of the electrolyte types, concentrations, and bulk pH (i.e., 0.1 M NaOH, 1 M neutral phosphate buffer, and 0.1 M Na_2SO_4). Therefore, the nonlinearity observed in the present plots was evidently a consequence of the electronic structure of the semiconductor itself (i.e., the existence of two donor levels) rather than any effect of the surface morphology or the electrolyte, or the partial potential drop within the Helmholtz layer.

Experimental apparatus used during RRDE analyses: A photographic image of the Nb:STO disk electrode components is presented in Figure S8a. A Nb:STO single crystal disk wafer having a diameter of 4 mm was attached to a custom-made stainless steel (SUS) body using indium soldering. The resulting assembly was subsequently sealed in a commercially-available Teflon spacer using an epoxy resin (Figure S8b). A cross-sectional diagram of this disk-spacer assembly is shown in Figure S8c. Finally, the assembly was loaded into a commercially-available Pt ring to prepare the rotating Pt ring-Nb:STO single crystal disk electrode used for the experimental work (Figure S8d).

The experimental setup employed during the RRDE trials is shown in Figure S9. The electrode prepared through the procedure described above was mounted on a commercially-available electrode rotator system. The photoanode disk portion was illuminated by the UV LED through the bottom of a Pyrex beaker that was positioned with a custom-made seat. The distance between the photoanode and the light source and the relative positions of both remained fixed throughout each experiment.

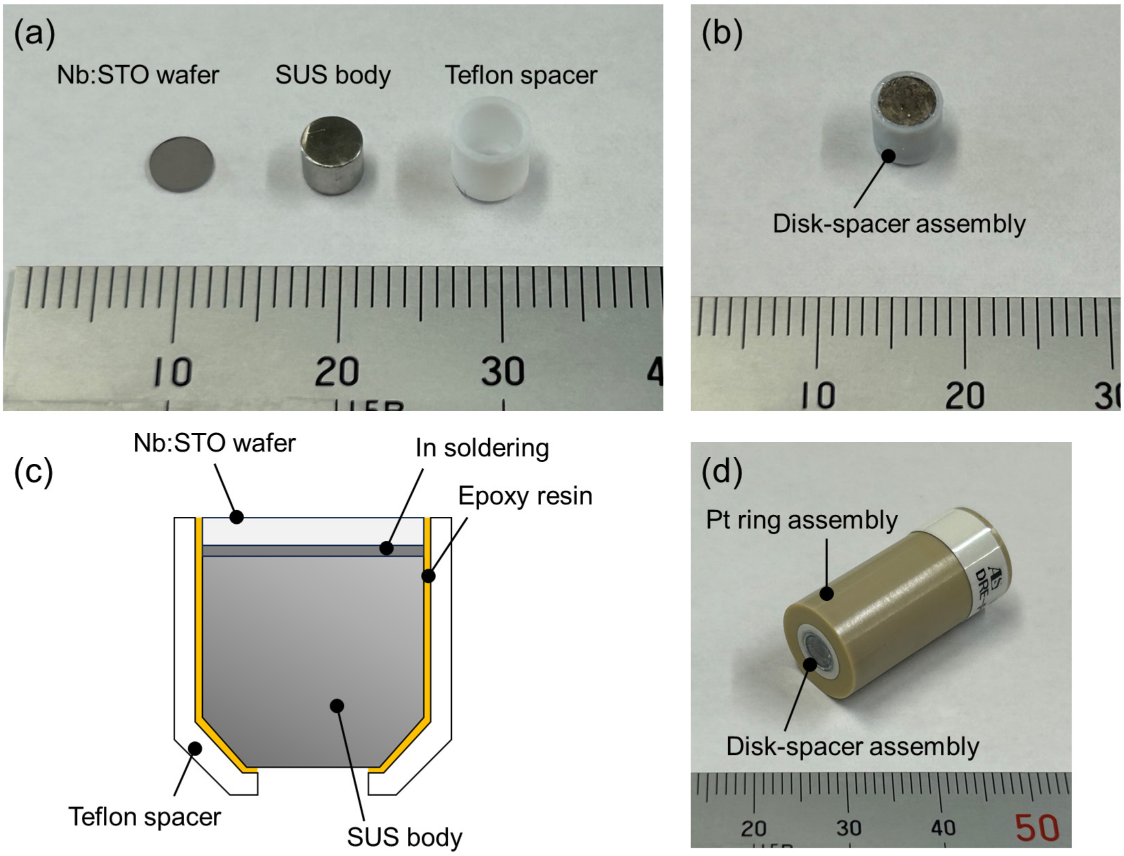

Figure S8. Photographic images of (a) the components used to prepare the Nb:STO disk and (b) the completed assembly consisting of the wafer, SUS body and Teflon spacer. (c) A cross-sectional drawing of the disk-spacer assembly. (d) The rotating Pt ring-Nb:STO single crystal disk electrode used during the RRDE experiments.

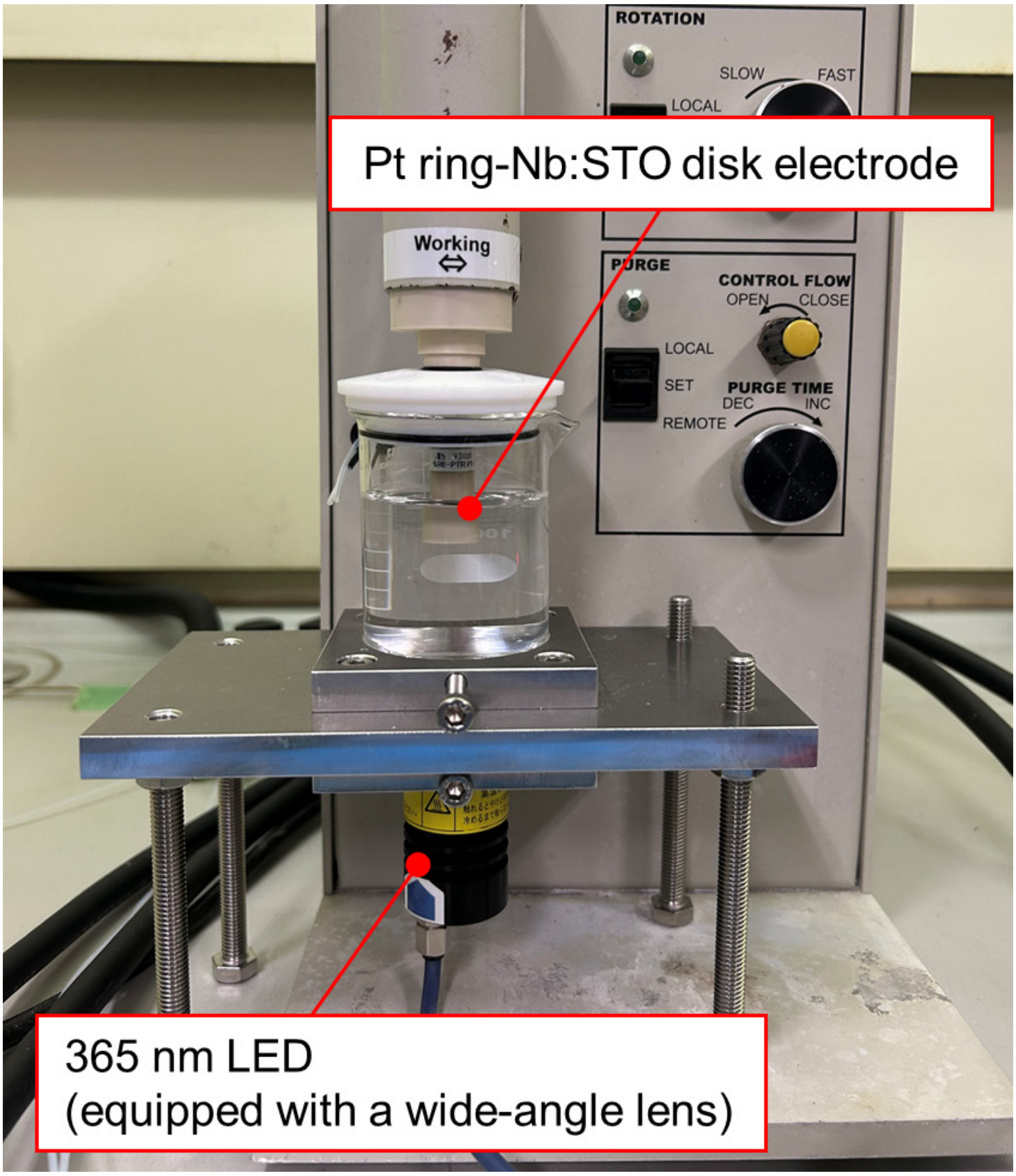

Figure S9. A photographic image of the experimental apparatus used during the RRDE experiments.

Discussion regarding detection of O_2 using the Pt ring electrode: To assess the detection of O_2 evolved at the disk electrode, it is important to carefully consider the O_2 collection efficiency at the ring electrode.[10,11] Using a relatively simple redox pair dissolved in a liquid medium ($[Fe(III)(CN)_6]^{3-}/[Fe(II)(CN)_6]^{4-}$), the collection efficiencies determined for the RRDE were consistent with the theoretical value calculated based on the electrode geometry.[18] The efficiency was also almost independent of the reaction rate (that is, the current density) at the disk electrode and the electrode rotation rate (Figures S10 and S11). In contrast, the O_2 collection efficiency was greatly affected by the current generated at the disk electrode, and by the electrode rotation rate and the type of electrolyte used (Figure S12). Because both the disk anodic photocurrent and the ring cathodic current (as presented in Figure 3a in the main manuscript) were quite low, it was unfortunately difficult to precisely determine the corresponding Faradaic efficiency. Nevertheless, a rough estimation was made based on the relationship between the O_2 collection efficiency and the disk current using the Pt ring-Pt disk electrode (Figure S13). From this estimation, it appears that a major portion of the photocurrent obtained several seconds after the initiation of light exposure could be attributed to the oxygen evolution reaction. More detailed discussions were provided below.

The ability of the Pt ring electrode to detect oxidant produced at the disk electrode during the RRDE trials was examined. The redox reaction of the $[Fe(III)(CN_6)]^{3-}/[Fe(II)(CN)_6]^{4-}$ pair was initially assessed as a model case of an ideal reversible one-electron process involving an outer-sphere electron transfer mechanism. CV data were acquired using a Pt RDE in a 0.1 M Na_2SO_4 electrolyte containing 1 mM $[Fe(III)(CN_6)]^{3-}$ and 1 mM $[Fe(II)(CN)_6]^{4-}$ while varying the rotational rate, with the results presented in Figure S10. When immersed in an electrolyte containing equimolar amounts of the reductant and oxidant, this Pt RDE generated sigmoidal CV curves that were symmetric with respect to the equilibrium potential. The anodic and cathodic diffusion limiting current values were almost identical and these values increased with increases in the electrode rotational rate. Note that the cathodic current originating from the reduction of $[Fe(III)(CN_6)]^{3-}$ was already limited by diffusion processes at an applied potential of 0 V vs. Ag/AgCl. Thus, the potential of the Pt ring electrode during the RRDE trials using $[Fe(II)(CN)_6]^{4-}$ as a reactant (as described in the following paragraph) was fixed at this value.

CV data were obtained from the Pt disk electrode together with data showing the effect of the disk electrode potential on the Pt ring current while varying the rotational rate. These experiments took place in an aqueous electrolyte containing 0.1 M Na_2SO_4 and 1 mM $[Fe(II)(CN)_6]^{4-}$ (Figure S11a). When using an electrolyte containing only the reductant, the onset of

the anodic current generated by the Pt disk electrode was shifted to a slightly negative potential compared with the $[Fe(III)(CN_6)]^{3-}/[Fe(II)(CN)_6]^{4-}$ equilibrium potential. In addition, the anodic current plateaued at a diffusion-limited value at positive applied potentials. In response to these anodic disk currents, the Pt ring generated cathodic currents. In this case, the ratio of the ring current to the disk current equaled the collection efficiency. It should also be noted that current values rather than current density values were used during these calculations. Efficiency values were calculated from the measured currents shown in Figure S11a and are plotted in Figure S11b as a function of the disk electrode potential. The dashed line in this plot indicates the theoretical value determined from the electrode geometry according to the equations,[18]

$$Collection\ efficiency = 1 - F\left(\frac{\alpha}{\beta}\right) + \beta^{\frac{2}{3}}\{1 - F(\alpha)\} - (1 + \alpha + \beta)^{\frac{2}{3}}\left\{1 - F\left(\frac{\alpha}{\beta}(1 + \alpha + \beta)\right)\right\},$$

$$\alpha = \left(\frac{r_2}{r_1}\right)^3 - 1,$$

$$\beta = \left(\frac{r_3}{r_1}\right)^3 - \left(\frac{r_2}{r_1}\right)^3$$

and

$$F(\theta) = \frac{3^{1/2}}{4\pi}\ln\left\{\frac{\left(1+\theta^{1/3}\right)^3}{1+\theta}\right\} + \frac{3}{2\pi}\arctan\left(\frac{2\theta^{1/3}-1}{3^{1/2}}\right) + \frac{1}{4},$$

where r_1, r_2 and r_3 are the disk radius, ring internal radius and ring outer radius, respectively, with values of 2, 2.5 and 3.5 mm, respectively, for the present Pt-Pt RRDE. In this manner, the theoretical collection efficiency was determined to be 0.42. In the case of the cyclic potential scans, the calculated collection efficiencies were close to the theoretical values regardless of the rotational rate, for relatively positive potentials at which the disk current was determined by diffusion processes. However, the efficiencies were greatly affected by the rotational rate, disk potential and disk current at negative potentials associated with charge transfer effects. Disk and ring current data as functions of time were obtained by applying various potentials to the Pt disk electrode and are summarized in Figure S11c. The collection efficiencies calculated from the steady-state current values in these chronoamperograms are also plotted in Figure S11d as a function of the disk electrode potential. Upon applying a constant potential rather than a cyclic scan, the collection efficiencies approached the theoretical value and were almost independent of the rotational rate, disk potential and disk current, even at relatively negative potentials. These almost ideal characteristics can possibly be attributed to the use of a relatively simple redox pair dissolved in a liquid medium.

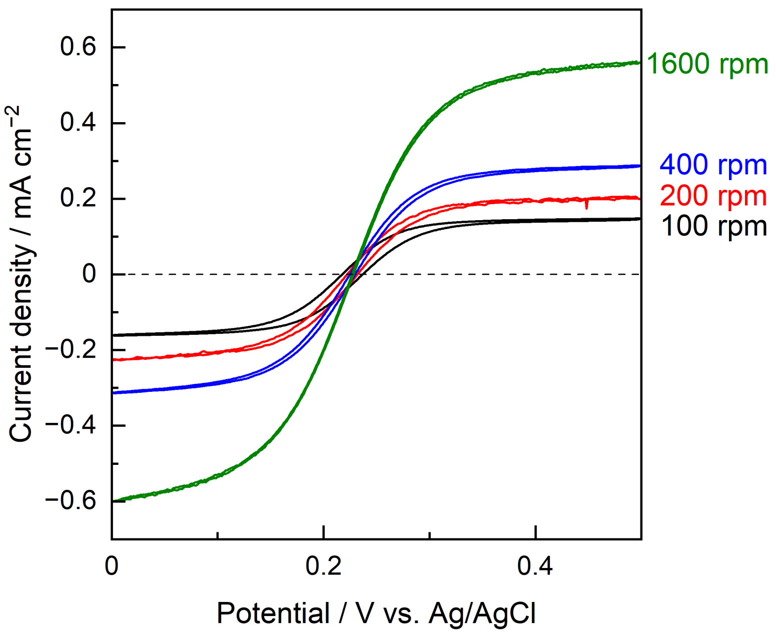

Figure S10. CV data obtained from a Pt RDE at various rotational rates in a 0.1 M Na_2SO_4 electrolyte containing 1 mM $[Fe(III)(CN_6)]^{3-}$ and 1 mM $[Fe(II)(CN)_6]^{4-}$ under Ar.

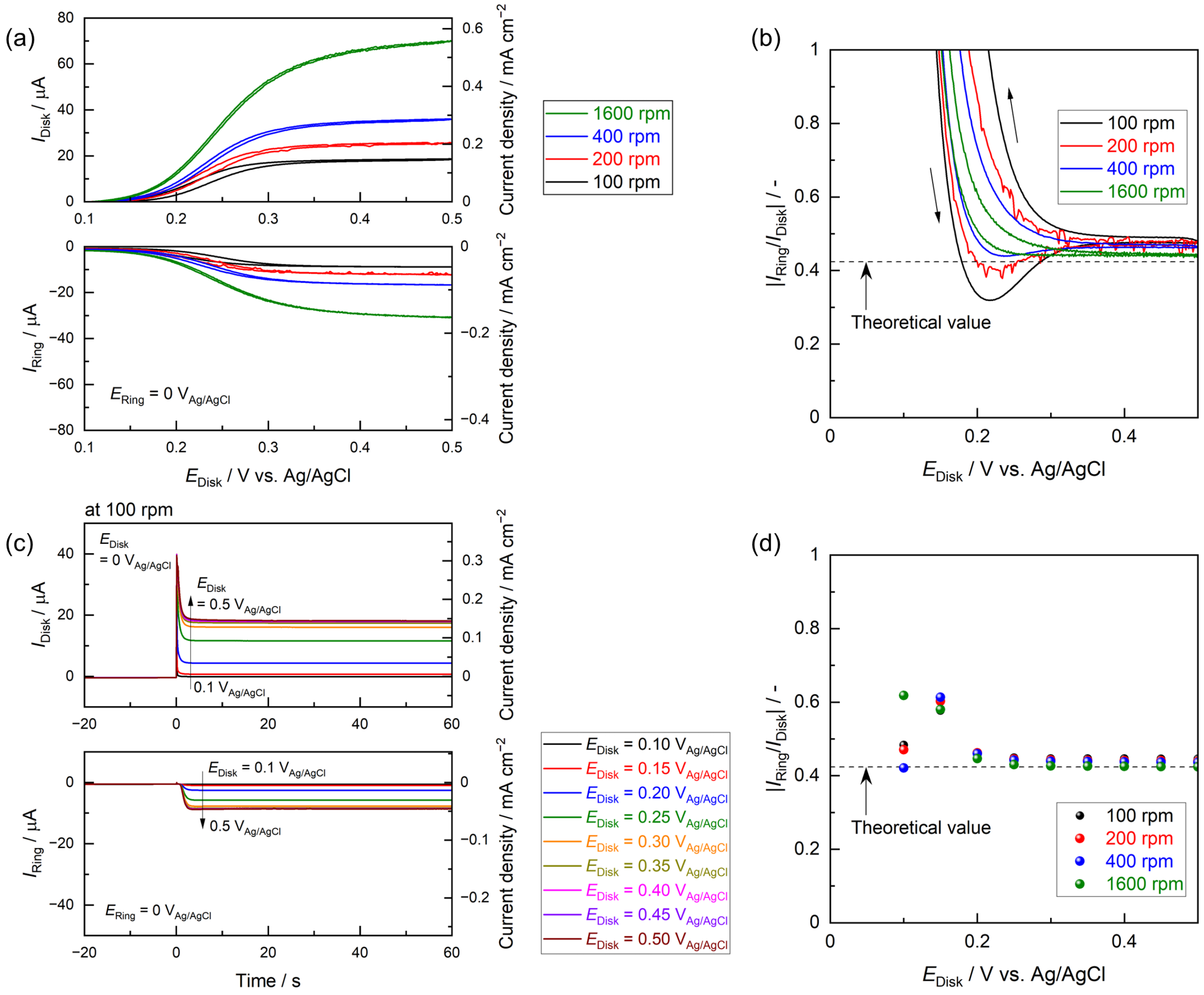

Figure S11. (a) CV data obtained from a Pt RDE and Pt ring current values acquired simultaneously, both as functions of the disk electrode potential in a 0.1 M Na_2SO_4 electrolyte containing 1 mM $[Fe(II)(CN)_6]^{4-}$ and (b) the corresponding calculated collection efficiencies. (c) Chronoamperograms acquired using Pt disk and Pt ring electrodes as generated by applying various potentials to the disk electrode and (d) the corresponding calculated collection efficiencies. A constant potential of 0 V vs. Ag/AgCl was applied to the Pt ring electrode and the trials were conducted under Ar.

The oxygen evolution reaction was evaluated using the Pt disk electrode while the oxygen reduction reaction was performed with the Pt ring electrode. During these experiments, the potential of the Pt ring electrode was fixed at 0.6 V_{RHE}. At this potential, oxygen is primarily reduced to water with only minimal formation of hydrogen peroxide.[19] The ring currents generated over time in conjunction with a constant Pt disk current in a Na_2SO_4 electrolyte are plotted as functions of time in Figure S12a. The collection efficiencies calculated from these data are also plotted as a function of the disk current in Figure S12b. The O_2 collection efficiencies were obviously smaller than the theoretical value determined from the electrode geometry. The Pt-Pt RRDE exhibited O_2 collection efficiencies almost independent of the disk current in the case that the Pt disk generated relatively high reaction rates, with current values on the order of several hundreds of microamperes. In contrast, relatively low oxygen evolution reaction rates significant decreased the O_2 collection efficiencies as the disk current was decreased. That is, the efficiency with which O_2 could be detected by the ring electrode was affected by the O_2 production rate at the disk electrode.[10,11] This unexpected phenomenon can possibly be ascribed to the low solubility of O_2 in the aqueous phase. In addition, both the oxygen evolution and reduction reactions are complicated multi-step processes involving multiple electron transfers and so were kinetically sluggish on the Pt electrocatalyst. In the case that the same experiments were conducted using the KPi electrolyte, the resulting collection efficiencies showed a similar effect of the disk current as was observed in the Na_2SO_4 electrolyte but were significantly lower. This effect could have resulted from the reduced solubility of O_2 in the concentrated KPi solution compared with the Na_2SO_4 solution.[20] The effects of the electrode rotational rate on the ring current with a constant current from the Pt disk in the Na_2SO_4 electrolyte are summarized in Figure S12c. The collection efficiencies calculated from these data are plotted as a function of the electrode rotational rate in Figure S12d. Interestingly, a higher rotational rate resulted in a more rapid response to changes in the oxygen concentration in the vicinity of the electrode surface but decreased the O_2 collection efficiency. These observations suggest that the O_2 collection efficiency was greatly modified by the magnitude of the current produced by the disk electrode, as well as by the electrode rotational rate and the type of electrolyte.

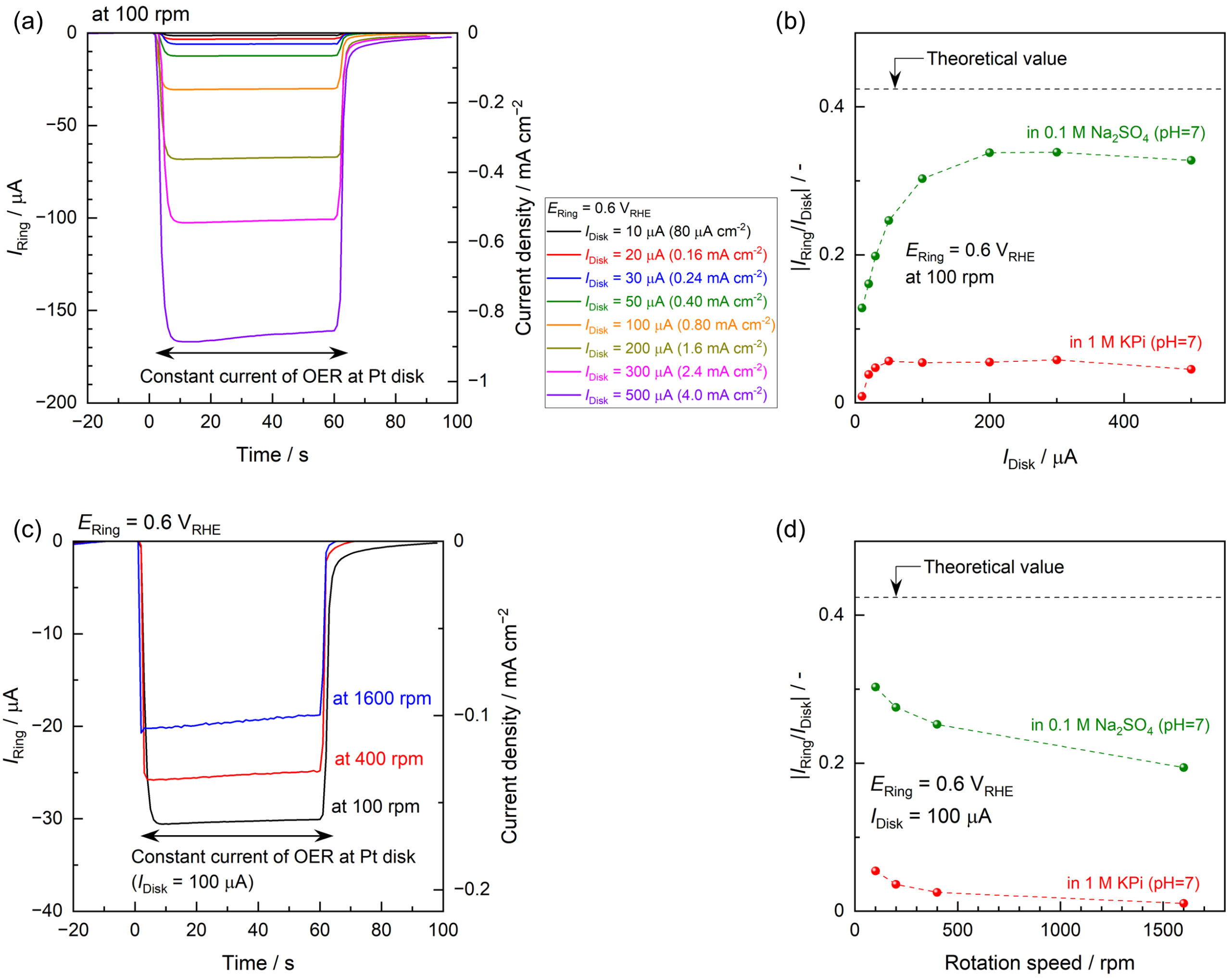

Figure S12. (a) Pt ring currents measured in conjunction with a constant Pt disk current in a neutral unbuffered electrolyte (0.1 M Na_2SO_4, adjusted to pH = 7) with a rotational rate of 100 rpm as functions of time and (b) the corresponding calculated collection efficiencies as a function of the disk current. (c) The ring current data acquired using various rotational rates as functions of time and (d) the corresponding collection efficiencies as a function of the rotational rate.

Attempts were made to use the RRDE to assess PEC oxygen evolution but the difficulties associated with determining the collection efficiency as described above were encountered. In addition, several oxidative by-products may have been generated at the disk electrode and could have affected the ring cathodic current. Specifically, various cations could have been released from the disk photoanode into the electrolyte due to photocorrosion, such that the Pt ring may have generated a cathodic current even in the case that the disk photoanode was not producing oxygen. The equilibrium potentials for possible reactions in the aqueous phase related to various cations originally involved the photoanode components (Nb, Sr and Ti) are listed below.[21]

	E° / V vs. SHE
$Sr^{2+} + 2e^- \rightleftarrows Sr$	−2.89
$Ti^{2+} + 2e^- \rightleftarrows Ti$	−1.63
$Nb^{3+} + 3e^- \rightleftarrows Nb$	−1.1
$Ti^{3+} + e^- \rightleftarrows Ti^{2+}$	−0.37
$TiO^{2+} + 2H^+ + e^- \rightleftarrows Ti^{3+} + H_2O$	0.100

During the present trials, the potential of the Pt ring electrode was fixed at 0.6 V_{RHE}, which is equivalent to −0.012 V vs. Ag/AgCl and thus to 0.187 V vs. a standard hydrogen electrode (SHE). The water-soluble chemicals containing Nb, Sr or Ti were not electrochemically reduced at this electrode potential. Therefore, the cathodic current generated by the Pt ring electrode during these measurements can be attributed solely to the oxygen reduction reaction.

Figure 3a in the main manuscript is again presented here as Figure S13, which plots the current values rather than current density values. These results were obtained with the RRDE rotating at 100 rpm and with potentials of 0 and 0.6 V_{RHE} applied to the Nb:STO disk and the Pt ring electrode, respectively. The disk and ring currents were respectively 3.5 and −0.6 µA following 2 s of irradiation but changed to 0.86 and −0.14 µA after 1 min, as shown by the arrows in the figure. The apparent collection efficiencies, I_{Ring}/I_{Disk}, for the Pt-Nb:STO RRDE were calculated to be in the range of 0.17 – 0.18. The O_2 collection efficiencies of the Pt-Pt RRDE ranged between 0.1 and 0.2 when the disk current values were relatively low, as demonstrated in Figure S12. It should be noted that, because both the disk anodic photocurrent and ring cathodic current (Figure S13) were quite low, it was unfortunately difficult to determine the corresponding Faradaic efficiency with certainty. Nevertheless, the O_2 collection efficiency of the Pt-Nb:STO RRDE under UV light was quite similar to the value obtained using the Pt-Pt RRDE. Hence, the photocurrent produced by the Nb:STO disk several seconds after initiating light exposure can be attributed to the oxygen evolution reaction.

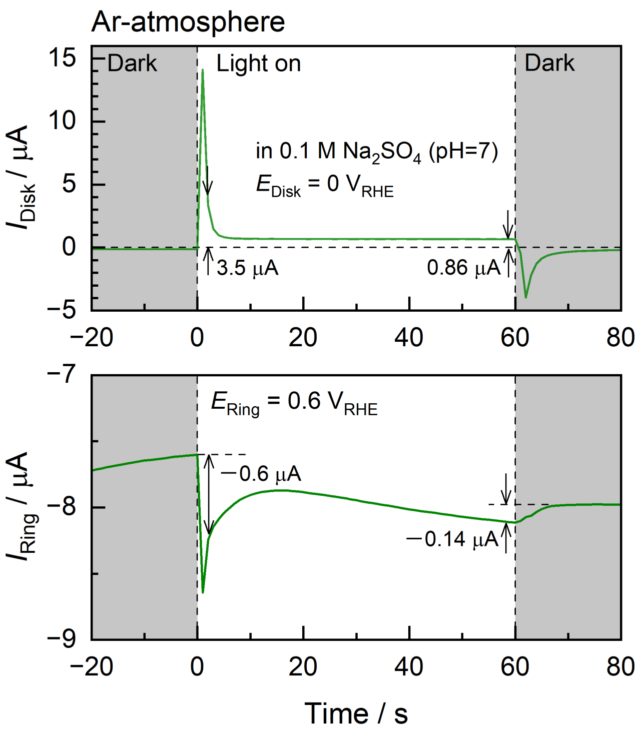

Figure S13. The disk photocurrent (at 0 V_{RHE}) and the simultaneously recorded ring current (at 0.6 V_{RHE}) obtained from a rotating Pt ring-Nb:STO single crystal disk electrode under Ar as functions of time. These trials employed a 100 rpm rotational rate for the RRDE and a 365 nm LED light source. This figure is reproduced from Figure 3a in the main manuscript. The disk and ring current values after 2 s or 1 min of light exposure are indicated by arrows.

Buffering capabilities of KPi solutions having different concentrations: Changes in the pH values of 0.1 M Na_2SO_4 solutions containing various concentrations of KH_2PO_4 with the addition of NaOH are summarized in Figure S14a. The buffer strength, β, of these solutions could be calculated as

$$\beta = \left(\frac{d\mathrm{pH}}{dn_{\mathrm{base}}}\right)^{-1}$$

where n_{base} is the moles of NaOH added. Based on the reciprocal of the derivatives of the titration curves, the effect of pH on buffer strength was calculated with the results presented in Figure S14b. The Na_2SO_4 solution did not exhibit any buffering effect at all at near-neutral pH values, whereas dilute KH_2PO_4 (with concentrations on the order of only several millimolar) showed buffering capacity. The buffer strength was also found to increase as the phosphate concentration increased, with the highest value obtained at a pH of approximately 6.7. This value reflects the pKa of the phosphoric acid. Additionally, the buffer strength of the Na_2SO_4 solution at pH values above 8 was found to gradually increase in response to increases in pH.

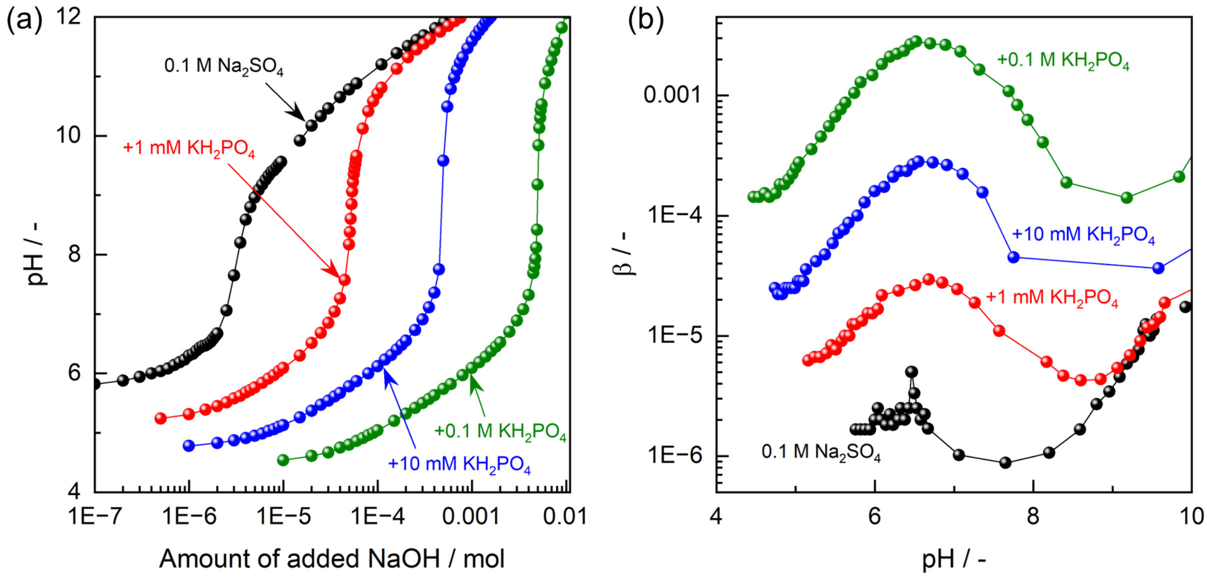

Figure S14. (a) The pH values of aqueous solutions containing 0.1 M Na_2SO_4 and various concentrations of KH_2PO_4 as functions of the moles of NaOH added. (b) Buffer strength as a function of the solution pH.

Effects of the KPi concentration on PEC properties: Current-potential curves and Mott-Schottky plots obtained using a Nb:STO photoanode in 0.1 M Na_2SO_4 solutions containing various concentrations of KPi (all above 5 mM) are compiled in Figure S15. As discussed in the main manuscript, even relatively low KPi concentrations (in the range of approximately 5 – 10 mM) suppressed the unique hysteresis in the CVs and Mott-Schottky plots in response to UV irradiation, depending on the potential scan direction. When the phosphate concentration was increased above 5 mM, the anodic photocurrent in the negative potential region in the current-potential curves gradually increased. That is, the point of maximum power in the curve was shifted to more negative potentials at higher photocurrents. At the same time, the Mott-Schottky plots obtained with UV light exposure no longer exhibited a plateau at negative potentials. As such, these plots had shapes similar to those acquired in highly alkaline solutions or in the absence of light. Hence, the denser KPi solutions provided current-potential curves and Mott-Schottky plots closer to the ideal behavior.

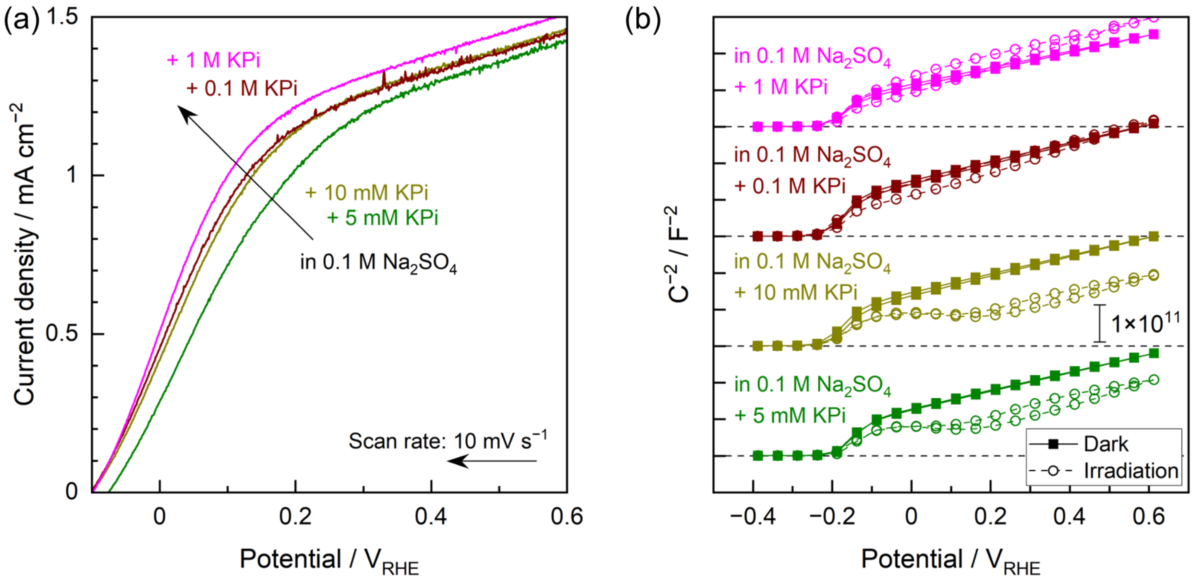

Figure S15. (a) Current-potential curves obtained using a Nb:STO single crystal photoanode in 0.1 M Na_2SO_4 electrolytes containing various concentrations of KPi with exposure to a 365 nm LED. The electrolytes were purged with Ar and vigorously stirred. (b) Mott-Schottky plots acquired in the absence of forced convection with and without UV irradiation.

Effects of the KPi concentration on oxygen evolution kinetics: Oxygen evolution reaction trials were performed using the Pt RDE in Na_2SO_4 electrolytes containing various concentrations of KPi, with the results presented in Figure S16. In the case that the unbuffered electrolyte containing only Na_2SO_4 was employed, the anodic current monotonically increased with increases in the applied potential above 2.1 V_{RHE}. These currents were only minimally affected by the electrode rotational rate and thus were attributable to the oxidation of water to produce oxygen. When a relatively low concentration of KPi was added to the electrolyte, an anodic current was observed at potentials more negative than 2 V_{RHE} but this current rapidly plateaued. Applying more positive potentials again increased the current and the plateau value of the current increased as the electrode rotational rate was increased, indicating a diffusion-limited process. The plateau current also increased along with the KPi concentration. Therefore, the anodic current observed at potentials more negative than the potential associated with water oxidation originated from oxygen evolution involving the KPi.[22] In such systems, oxygen evolution proceeds in conjunction with the production of protonated buffering species rather than protons, and so the diffusion of these protonated species governs the overall reaction rate.[22] The recovery of the anodic current at more positive potentials reflects a reactant switching effect.[22] Increasing the KPi concentration to approximately 0.1 M caused the anodic current to appear at potentials more negative than 2 V_{RHE} and to monotonically increase as the electrode potential increased. Because the KPi concentration was increased to a sufficiently high value, the anodic current was no longer governed by the diffusion of the anion species in this reaction rate range. Therefore, the ideal PEC behavior of the photoanode in the dense KPi electrolyte (as described in the previous section) should be attributed not only to a reduction of the local pH gradient but also to an acceleration of the oxygen evolution reaction kinetics.

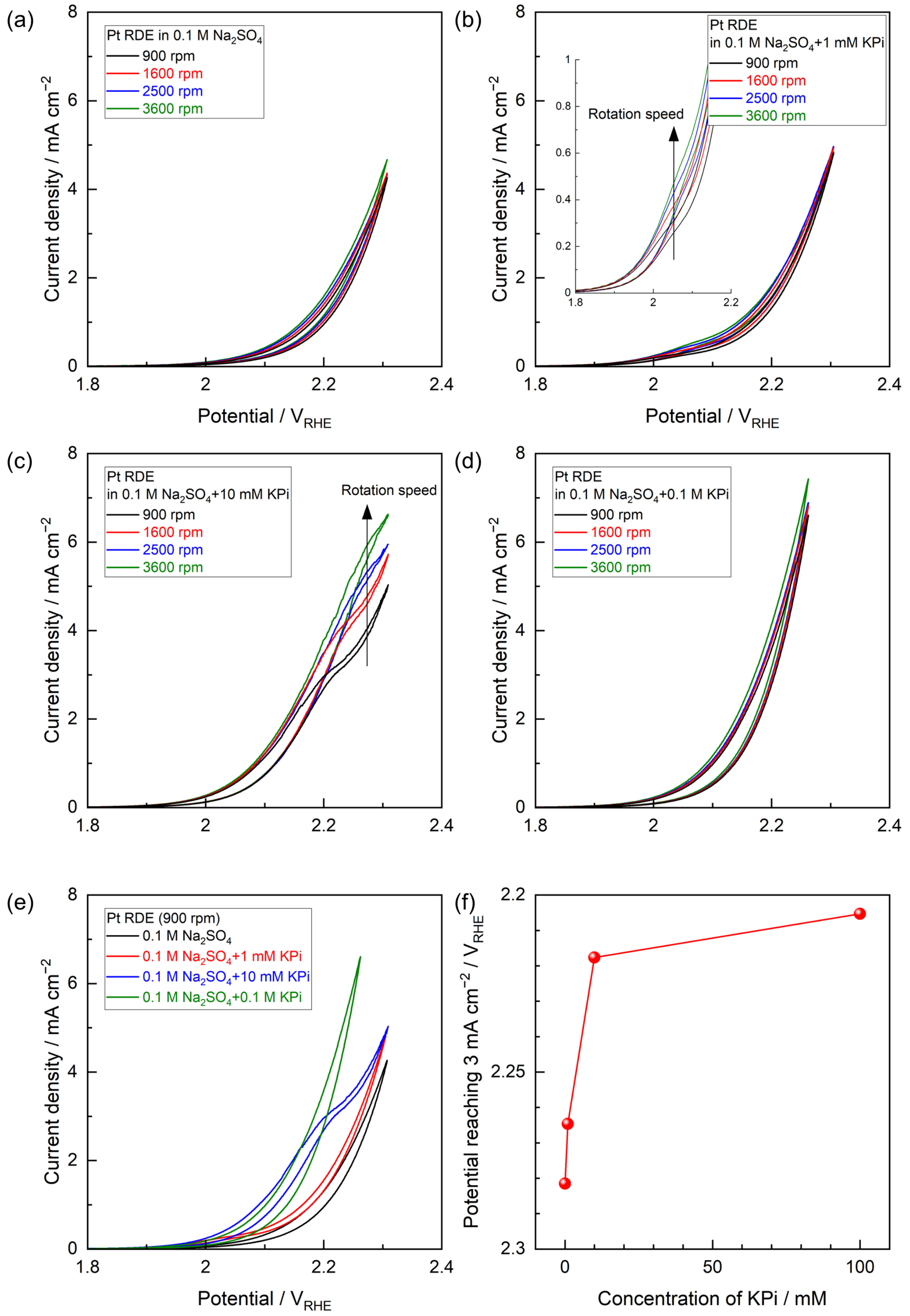

Figure S16. (a – e) CV data obtained using a Pt RDE with various rotational rates in 0.1 M Na_2SO_4 electrolytes containing different concentrations of KPi and (f) the overpotentials required to obtain an anodic current of 3 mA cm^{-2} as a function of the KPi concentration.

Effects of the electrolyte pH on the shapes of Mott-Schottky plots: The Mott-Schottky plots obtained in unbuffered electrolytes with various pH values with and without UV irradiation (originally presented as Figures 5d and 5e in the main manuscript) are again compared without an offset in the y-axis direction in Figure S17. Under dark conditions, the plots monotonically shifted to negative potentials in response to increases in pH while maintaining almost the same slope and shape (Figure S17a). This phenomenon reflects an ideal Nernst-like shift of the band-edge potentials. In contrast, it was evident that, when the electrolytes with pH values ranging from 3 to 11 were employed, the shapes of the Mott-Schottky plots obtained with UV irradiation were almost identical at potentials more positive than approximately −0.5 V vs. Ag/AgCl irrespective of the bulk pH (Figure S17b). This insensitivity of the Mott-Schottky plot slopes to the electrolyte pH can be ascribed to undesirable band-edge unpinning relative to a RHE resulting from the relatively weak buffering capability in this pH region.

When weakly alkaline solutions with pH values of 8 – 10 were employed, the Mott-Schottky plots produced using both anodic and backward cathodic scans under UV irradiation plateaued at relatively negative potentials in the vicinity of the onset potential (Figure S18a). That is, irrespective of the potential scan direction, the plots exhibited inflection points. The C^{-2} values within the plateau region during the cathodic scans gradually increased in response to the increases in pH. The Mott-Schottky plots acquired in the pH 11 electrolyte under UV light eventually exhibited less hysteresis but still plateaued within the potential region ranging from −0.9 to −0.6 V vs. Ag/AgCl (Figure 5e in the main manuscript). This trend was similar to that observed when employing a low concentration of KPi, as shown in Figure 4. Specifically, increasing of pH in the weakly alkaline range caused the semiconductor/electrolyte interface to gradually approach the ideal band-edge pinning conditions as the buffering capability of the electrolyte was slowly increased. However, even the pH 11 electrolyte was unable to completely suppress the undesirable positive shift of the band-edges under UV irradiation, despite the absence of the unique hysteresis effect in the CV data.

Two slopes in the vicinity of the onset potential associated with the cathodic scan Mott-Schottky plots, as well as a slope produced around the onset of the anodic scan, acquired under UV irradiation in weakly alkaline electrolytes are shown as solid lines in Figure S18a. Here, it should be noted that the flat-band potentials within this pH region with exposure to UV light (see Figure 5f in the main manuscript) were estimated from linear fitting of the data produced at relatively positive potentials ranging from −0.8 to −0.6 V vs. Ag/AgCl. In the case that the shorter linear portion at relatively negative potentials close to −1 V vs. Ag/AgCl was employed to calculate the flat-band

potentials under these conditions, the effect of pH on the flat-band potentials could be ascertained, as presented in Figure S18b. It is evident that the flat-band potentials determined from the slopes at relatively negative potentials were pinned at an almost constant value reflecting the pH of the bulk electrolyte under weakly alkaline conditions, regardless of the potential scan direction or light exposure. Consequently, the gradual improvement in the buffering capability of the electrolyte facilitated the return of flat-band potentials during PEC oxygen evolution to the original values reflecting the bulk pH, but only in the case that the PEC reaction rate was low.

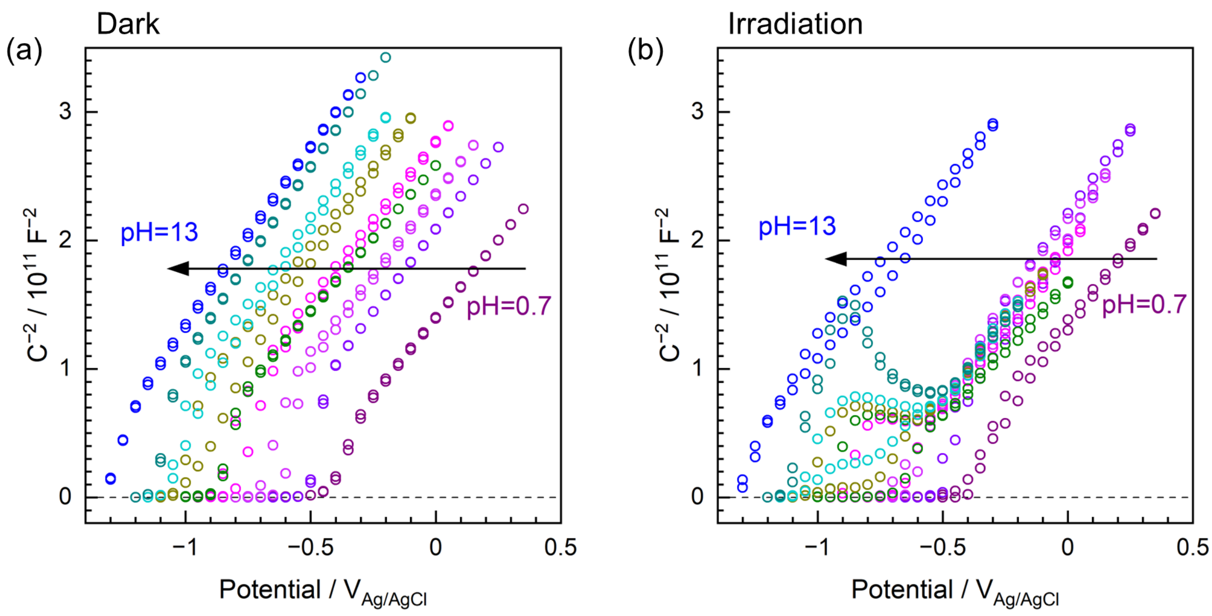

Figure S17. Mott-Schottky plots acquired in electrolytes with various pH values using cyclic potential scans (a) without and (b) with UV irradiation. These figures were reconstructed from Figures 5d and 5e in the main manuscript such that the plots could be compared without any offset in the *y*-axis direction.

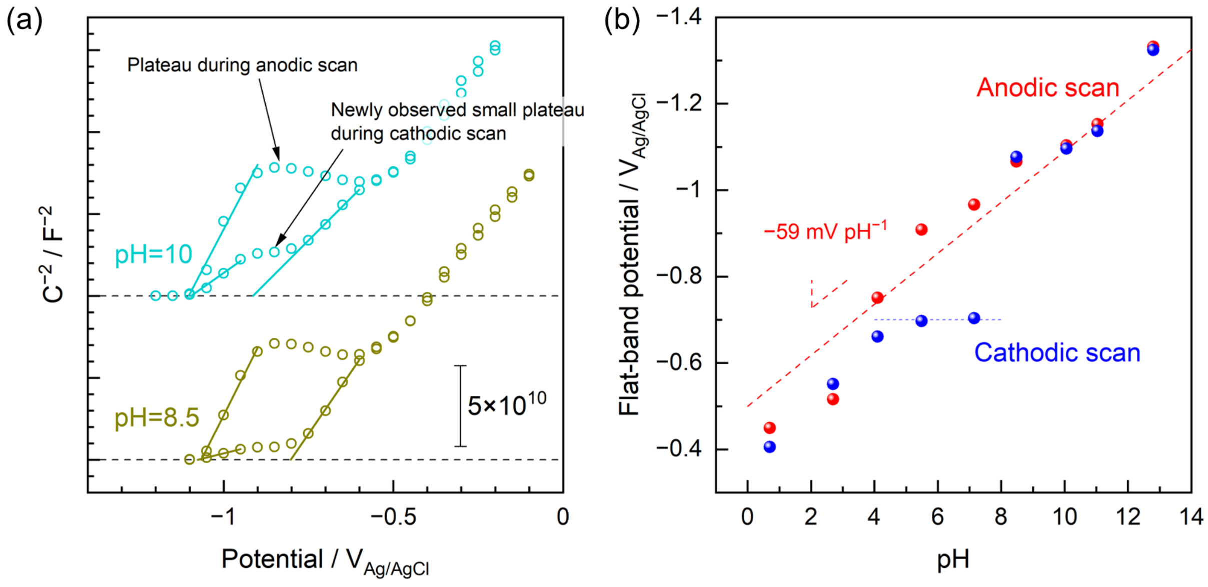

Figure S18. (a) Mott-Schottky plots acquired in unbuffered electrolytes containing 0.1 M Na_2SO_4 with pH values of 8.5 and 10. The linear fittings used to determine the flat-band potentials are presented as solid lines. (b) Apparent flat-band potentials estimated from anodic and cathodic scans under UV irradiation as functions of the electrolyte pH. Note that the two data points representing pH values of 8.5 and 10 in the blue plot are different from the data presented in Figure 5f in the main manuscript.

Effects of the electrolyte buffering capabilities on the PEC properties of various particulate photoanodes: We evaluated the PEC properties of the particulate TiO_2 photoanode in the neutral buffered or unbuffered electrolyte as summarized in Figure S19. The anodic photocurrent acquired during these experiments should be derived from oxygen evolution reaction. The hysteresis behavior similar to the case of Nb:STO single crystalline photoanode depending on the buffering capability of electrolytes was certainly observed. That is, the CV acquired in the buffered electrolyte exhibited little hysteresis, whereas the obvious hysteresis around the onset potential for the case of the neutral unbuffered electrolyte. It should be noted that the fill-factors of the CVs obtained using the particulate TiO_2 photoanode were lower than the case of single crystalline Nb:STO. This may simply reflect the sluggish kinetics of the physical processes inside the TiO_2 particles during the PEC reaction compared to the Nb:STO single crystal.

Furthermore, we also assessed the PEC properties of the particulate TiO_2 or WO_3 photoanodes for methanol oxidation reaction as shown in Figure S20. WO_3 is a well-known example of the visible-light-responsive oxide photocatalyst. Methanol oxidation reaction, which is usually employed as a sacrificial test reaction, involves the proton-coupled electron transfer process[23] but proceeds electrochemically easier than the oxygen evolution reaction. The CVs acquired in the neutral buffered or unbuffered electrolyte containing methanol also exhibited the hysteresis behavior similar to the case of oxygen evolution reaction performed by Nb:STO single crystal depending on the buffering capability of electrolytes. The important point is that these hysteresis behaviors were observed regardless of the choice of photoanodes (Nb:STO, TiO_2, or WO_3). Therefore, it can be concluded that the present unique hysteresis behavior during the PEC reaction involving the proton-coupled electron transfer should be mainly governed by the transient physicochemical phenomena inside the electrolyte and thus be essentially expected regardless of the choice of photoanode materials.

However, it has been well-known that the sulfide-based materials usually show different surface chemistry from the oxide materials and thus sometimes do not follow the ideal Nernst-like behavior. For instance, band-edge potentials of CdS were reported to be almost independent of the bulk electrolyte pH up to the weak alkaline region. In such cases, the dependence of flat-band potentials on the local pH variations during the PEC reaction may differ from the case of the metal oxide photoanodes reported in the present study, although CdS is basically unable to produce oxygen from water due to photocorrosion.

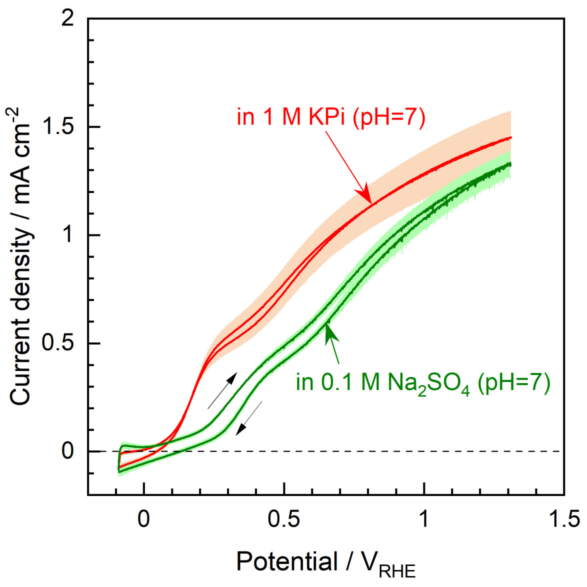

Figure S19. CV data obtained using a particulate TiO_2 photoanode in 0.1 M Na_2SO_4 or 1 M KPi upon exposure to a 365 nm LED and applying a scan rate of 10 mV s^{-1}. Each electrolyte was purged with Ar and vigorously stirred. The shaded areas around each plot indicate the standard error. The black arrows indicate the potential scan direction.

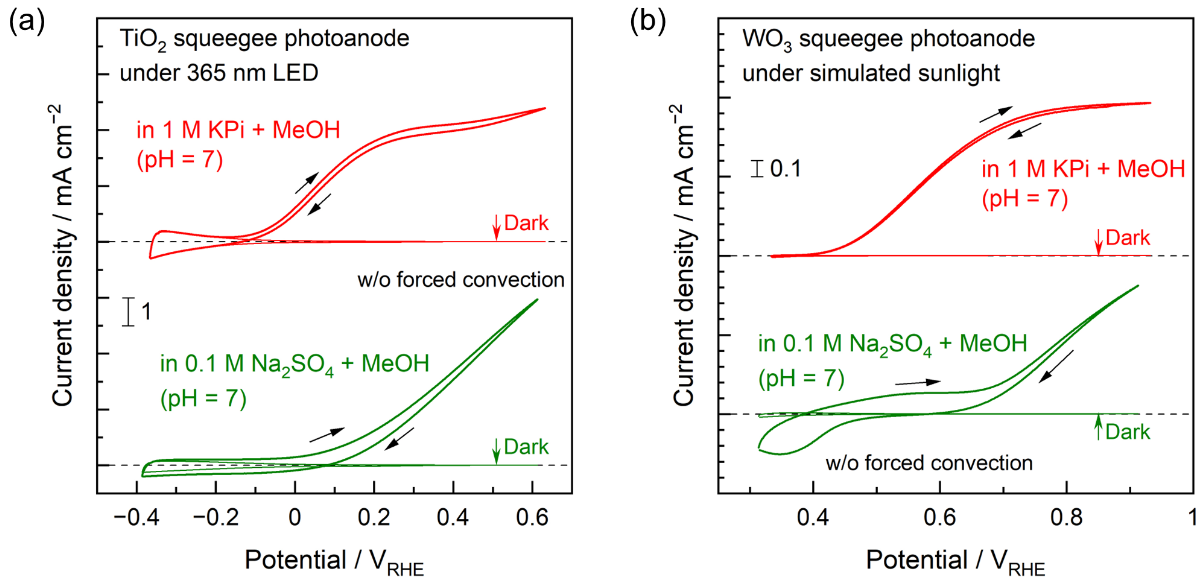

Figure S20. CV data obtained using (a) a particulate TiO_2 photoanode under illumination of 365 nm LED and (b) a particulate WO_3 photoanode under simulated sunlight (AM 1.5G) in 0.1 M Na_2SO_4 or 1 M KPi applying a scan rate of 10 mV s^{-1}. Each electrolyte was purged with Ar and vigorously stirred. The black arrows indicate the potential scan direction.

References

1. T. Takata, J. Jiang, Y. Sakata, M. Nakabayashi, N. Shibata, V. Nandal, K. Seki, T. Hisatomi and K. Domen, *Nature*, 2020, **581**, 411.
2. Y. Kageshima, H. Momose, F. Takagi, S. Fujisawa, T. Yamada, K. Teshima, K. Domen and H. Nishikiori, *Sustainable Energy Fuels*, 2021, **5**, 4850.
3. K. Sivula, *ACS Energy Letters*, 2021, **6**, 2549.
4. Y. Kageshima, H. Wada, K. Teshima and H. Nishikiori, *Appl. Catal. B*, 2023, **327**, 122431.
5. H. Kumagai, T. Minegishi, N. Sato, T. Yamada, J. Kubota and K. Domen, *J. Mater. Chem. A*, 2015, **3**, 8300.
6. B. H. Loo, K. W. Frese Jr. and S. R. Morrison, *Appl. Surf. Sci.*, 1981, **8**, 290.
7. J. R. Wilson and S.-M. Park, *J. Electrochem. Soc.*, 1982, **129**, 149.
8. C. A. Koval and R. Torres, *J. Am. Chem. Soc.*, 1993, **115**, 8368.
9. B. Su, Y. Ma, Y. Du and C. Wang, *Electrochem. Commun.*, 2009, **11**, 1154.
10. G. Li, L. Anderson, Y. Chen, M. Pan and P.-Y. A. Chuang, S*ustainable Energy Fuels*, 2018, **2**, 237.
11. J. G. Vos and M. T. M. Koper, *J. Electroanal. Chem.*, 2019, **850**, 113363.
12. M. Abdullah, G. K.-C. Low and R. W. Matthews, *J. Phys. Chem.*, 1990, **94**, 6820.
13. A. Linz Jr, *Phys. Rev.*, 1953, **91**, 753.
14. K. G. Rana, V. Khikhlovskyi and T. Banerjee, *Appl. Phys. Lett.*, 2012, **100**, 213502.
15. J. H. Kennedy and K. W. Frese Jr., *J. Electrochem. Soc.*, 1978, **125**, 723.
16. S. Takata, Y. Miura and Y. Matsumoto, *Phys. Chem. Chem. Phys.*, 2014, **16**, 24784.
17. A. Hankin, F. E. Bedoya-Lora, J. C. Alexander, A. Regoutz and G. H. Kelsall, *J. Mater. Chem. A*, 2019, **7**, 26162.
18. A. M. Zimer, M. Medina da Silva, E. G. Machado, H. Varela, L. H. Mascaro and E. C. Pereira, *Anal. Chim. Acta*, 2015, **897**, 17.
19. O. Antoine and R. Durand, *J. Appl. Electrochem.*, 2000, **30**, 839.
20. T. Naito, T. Shinagawa, T. Nishimoto and K. Takanabe, *ChemSusChem*, 2022, **15**, e202102294.
21. A. J. Bard, R. Parsons and J. Jordan, *Standard Potentials in Aqueous Solution*, Marcel Dekker, Inc., New York, 1985.
22. T. Shinagawa, M. T.-K. Ng and K. Takanabe, *ChemSusChem*, 2017, **10**, 4155.
23. M. J. S. Farias, W. Cheuquepán, A. A. Tanaka and J. M. Feliu, *ACS Catal.*, 2020, **10**, 543.
